# Reconstruction of lncRNA-miRNA-mRNA network based on competitive endogenous RNA reveals functional lncRNAs in skin cutaneous melanoma

**DOI:** 10.1186/s12885-020-07302-5

**Published:** 2020-09-29

**Authors:** Junyou Zhu, Jin Deng, Lijun Zhang, Jingling Zhao, Fei Zhou, Ning Liu, Ruizhao Cai, Jun Wu, Bin Shu, Shaohai Qi

**Affiliations:** 1 Department of Burn, The First Affiliated Hospital, Sun yat-sen University, Guangzhou, Guangdong 510080 People’s Republic of China; 2 Department of Radiation Oncology, Cancer Center of Guangzhou Medical University, Guangzhou, Guangdong 510095 People’s Republic of China

**Keywords:** Human skin cutaneous melanoma, lncRNA, Competitive endogenous RNA, *MALAT1*, *LINC00943*, *LINC00261*, miRNA

## Abstract

**Background:**

Human skin cutaneous melanoma is the most common and dangerous skin tumour, but its pathogenesis is still unclear. Although some progress has been made in genetic research, no molecular indicators related to the treatment and prognosis of melanoma have been found. In various diseases, dysregulation of lncRNA is common, but its role has not been fully elucidated. In recent years, the birth of the “competitive endogenous RNA” theory has promoted our understanding of lncRNAs.

**Methods:**

To identify the key lncRNAs in melanoma, we reconstructed a global triple network based on the “competitive endogenous RNA” theory. Gene Ontology and KEGG pathway analysis were performed using DAVID (Database for Annotation, Visualization, and Integration Discovery). Our findings were validated through qRT-PCR assays. Moreover, to determine whether the identified hub gene signature is capable of predicting the survival of cutaneous melanoma patients, a multivariate Cox regression model was performed.

**Results:**

According to the “competitive endogenous RNA” theory, 898 differentially expressed mRNAs, 53 differentially expressed lncRNAs and 16 differentially expressed miRNAs were selected to reconstruct the competitive endogenous RNA network. *MALAT1, LINC00943,* and *LINC00261* were selected as hub genes and are responsible for the tumorigenesis and prognosis of cutaneous melanoma.

**Conclusions:**

*MALAT1, LINC00943,* and *LINC00261* may be closely related to tumorigenesis in cutaneous melanoma. In addition, *MALAT1* and *LINC00943* may be independent risk factors for the prognosis of patients with this condition and might become predictive molecules for the long-term treatment of melanoma and potential therapeutic targets.

## Background

Human skin cutaneous melanoma (SKCM) is the most common and dangerous type of skin tumour [1, 2]. Worldwide, approximately 232,000 (1.7%) cases of cutaneous melanoma are reported among all newly diagnosed primary malignant cancers, and this disease results in approximately 55,500 cancer deaths (0.7% of all cancer deaths) [1, 3]. The incidence of melanoma in Australia, New Zealand, Norway, Sweden, the UK, and the USA from 1982 to 2011 has shown increases of approximately 3% annually and will further increase until 2022 [3]. In 2015, there were 3.1 million people with melanoma, resulting in 59,800 deaths [4]. Nevertheless, 95,710 cases of melanoma in situ will be newly diagnosed in 2020 [5]. The high incidence and high mortality of melanoma indicate that researchers must further study this disease. Although some achievements have been made in the genetic research of melanoma, markers related to diagnosis and treatment are needed.

Tumorigenesis often results from aberrant transcriptomes, including aberrant levels of coding RNA and noncoding RNA [6–8]. It has been proven that lncRNAs have various effects, including regulation of gene transcription, post-transcriptional regulation and epigenetic regulation [9–12]. In addition, dysregulation of lncRNAs has been observed in various diseases [13–16]. Unfortunately, the functions of lncRNAs are more difficult to identify than those of coding RNAs. Until now, only a few lncRNAs have been identified as crucial factors in the tumorigenesis and development of melanoma, including ZNNT1, THOR and SAMMSON [14, 15, 17]. Thus, how to locate them and define their functions is a challenge of current research.

The effect of miRNAs on malignancies has been verified in many ways. Studies have suggested that lncRNAs can regulate miRNA abundance by binding and sequestering them [18]. Thus, we aimed to study the function of lncRNAs by studying the interactions among lncRNAs, mRNAs and miRNAs. In 2011, the competitive endogenous RNA (ceRNA) hypothesis proposed a novel regulatory mechanism between noncoding RNA and coding RNA [19–21]. This theory indicated that any RNA transcript harbouring miRNA-response elements (MREs) can sequester miRNAs from other targets sharing the same MREs and thereby regulate their expression [19–21]. That is, the RNA transcripts that can be cross regulated by each other can be biologically predicted according to their common MREs [20, 22]. Evidence has shown that ceRNAs exist in several species and contexts and might play an important role in various biological processes, such as tumorigenesis [21]. Systematic analysis of the ceRNA network has been performed in multiple tumours, such as gastric cancer, bladder cancer, and ovarian cancer, contributing to a better understanding of tumorigenesis and facilitating the development of lncRNA-directed diagnostics and therapeutics against this disease [23–25]. Unfortunately, however, such functional interactions have not yet been elucidated in melanoma.

**Fig. 1:**
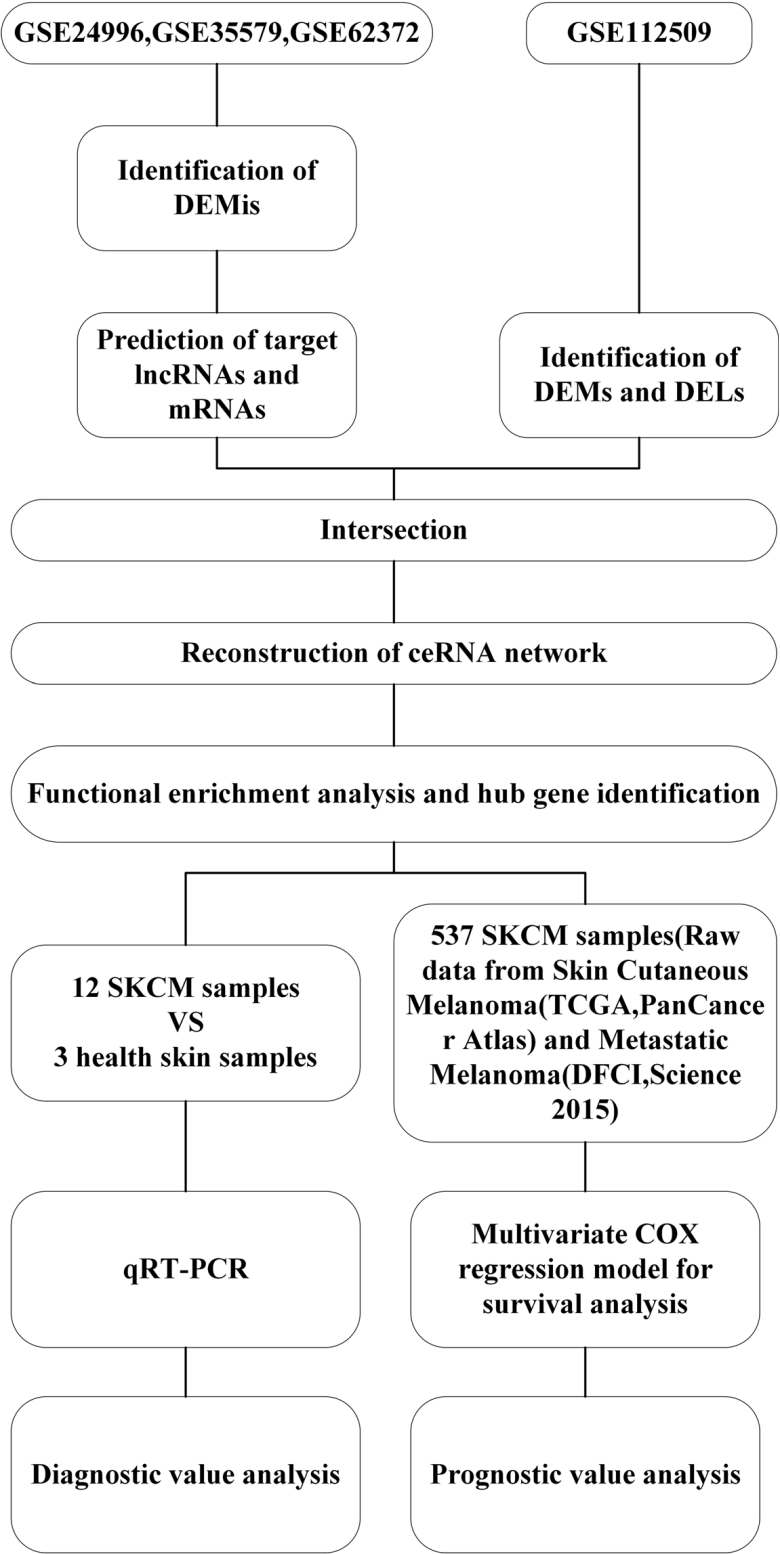
Study flow of this study

In this study, we used bioinformatics methods to construct the ceRNA network of cutaneous melanoma and to identify the key lncRNAs involved in melanomagenesis. Through the reconstruction of a ceRNA network, we identified and verified that the key ceRNA molecules play a crucial role in the tumorigenesis and prognosis of SKCM. (Work flow was shown in Fig. 1).

## Methods

### Raw data

Human melanoma miRNA expression data were downloaded from the NCBI GEO database (GEO (http://www.ncbi.nlm.nih.gov/geo) [26], including GSE24996, GSE35579, and GSE62372, which are array-based datasets. The GSE24996 dataset consists of 8 benign nevus tissue samples and 23 primary melanoma tissue samples. The GSE35579 dataset consists of 11 benign nevus tissue samples and 20 primary melanoma tissue samples. The GSE62372 dataset consists of 9 benign nevus tissue samples and 92 primary melanoma tissue samples. mRNA and lncRNA expression data were also downloaded from the NCBI GEO database (GSE112509), which is a sequence-based dataset. The GSE112509 dataset consists of 23 benign nevus tissue samples and 57 primary melanoma tissue samples.

### Identification of DEMis, DELs and DEMs

For identification of the differentially expressed miRNAs (DEMis) between primary melanoma and benign nevus samples, “R” (version 3.4.2, https://www.r-project.org/) [27] was used with the “limma” package after normalization [28]. For identification of the differentially expressed lncRNAs (DELs) and mRNAs (DEMs) between primary melanoma and benign nevus samples, “R” (version 3.4.2, https://www.r-project.org/) [27] was used with the “DESeq2” package [29]. The DEMis, DELs and DEMs were selected according to |log_2_FC| > 1 and adjusted *P*-value < 0.05.

### Prediction of target lncRNAs and mRNAs

For prediction of the target lncRNAs and mRNAs through DEMis, starBase (starbase.sysu.edu.cn) was used in our study [30]. Multiple lncRNA/mRNA-predicting programmes (PITA, RNA22, miRmap, DIANA-microT, miRanda, PicTar and TargetScan) were used in starBase [30]. For accuracy, only when the target mRNA was predicted in at least four predicted programmes on starBase would it be chosen as the predicted target mRNA. Then, these predicted target lncRNAs and mRNAs were merged with DEMs and DELs, respectively.

### Reconstruction of the ceRNA network

The ceRNA network was reconstructed based on ceRNA theory [20] and as follows: (1) Expression correlation between DELs and DEMs was evaluated using the Pearson correlation coefficient (PCC). The DEL-DEM pairs with PCC > 0.4 and *P*-value < 0.01 were considered coexpressed lncRNA-mRNA pairs. (2) Both lncRNAs and mRNAs in the pairs were negatively correlated with their common miRNAs. (3) The ceRNA network was reconstructed and visualized using Cytoscape (version 3.7.1, https://cytoscape.org/) [31, 32].

### Functional enrichment analysis

For functional enrichment, Gene Ontology (GO) biological process (BP), cell component (CC), molecular function (MF) and Kyoto Encyclopedia of Genes and Genomes (KEGG) pathway analysis of mRNAs in the ceRNA network were performed using DAVID (version 6.8, https://david.ncifcrf.gov/) [33, 34].

### Hub gene selection and reconstruction of key ceRNA subnetworks

To reconstruct our key ceRNA subnetwork, we first selected hub genes according to the node degrees of the ceRNA network we reconstructed above by calculating the number of lncRNA-miRNA and miRNA-mRNA pairs. For these key lncRNAs, GO-BP, GO-CC, GO-MF and KEGG pathway annotation were performed according to their first mRNA neighbours by using DAVID (version 6.8, https://david.ncifcrf.gov/) [33, 34].

### Sample selection for qRT-PCR validation

To validate findings in the ceRNA network, we selected the top three hub genes to determine their expression in cutaneous melanoma and skin tissues. Twelve patients with cutaneous melanoma and three healthy patients were included in this study. The study protocol was approved by the Ethics Committee of The First Affiliated Hospital, Sun Yat-sen University. All patients provided written informed consent in compliance with the code of ethics of the World Medical Association (Declaration of Helsinki). The eligible patients for this study had to meet the following criteria: (1) histologically confirmed as melanoma; (2) received no radiotherapy, chemotherapy or biotherapy before surgery. The exclusion criteria were as follows: (1) previous malignancies; (2) concomitant malignancies; (3) serious active infection; and (4) pregnancy or lactation.

Eligible cutaneous melanoma patients were from The First Affiliated Hospital, Sun Yat-sen University (Guangzhou, Guangdong, China) or the Cancer Center of Guangzhou Medical University (Guangzhou, Guangdong, China). Each tumour sample was matched with adjacent apparently normal tissues removed during the same operation. Frozen sections were made from these tissues and examined by at least three pathologists. The clinicopathological features of twelve skin cutaneous melanoma patients (51.67 ± 14.57 years old) for qRT-PCR validation are shown in Table 1. Three healthy patients from The First Affiliated Hospital, Sun Yat-sen University (Guangzhou, Guangdong, China) were included in this study. These patients were scheduled to undergo split-thickness skin grafting due to deep partial burn wounds. Each normal skin sample was obtained from the donor site. All the samples were frozen immediately after the operation and were stored in liquid nitrogen until RNA isolation.

**Table 1:** The clinicopathological features of twelve SKCM patients for qRT-PCR validation

Patients ID	Pathological diagnosis	TNM	Stage^a^
001	SKCM	T3AN1AM0	IIIB
002	SKCM	T3AN0M0	IIA
003	SKCM	T3BN0M0	IIB
004	SKCM	T2AN0M0	IA
005	SKCM	T1AN0M0	IA
006	SKCM	T1AN0M0	IA
007	SKCM	T2BN0M0	IIA
008	SKCM	T1AN0M0	IA
009	SKCM	T4BN2AM0	IIIC
010	SKCM	T2BN0M0	IIA
011	SKCM	T3AN0M0	IIA
012	SKCM	T3BN0M0	IIB

Abbrevations: *SKCM* Skin cutaneous melanoma; *TNM* Tumor node metastasis

^a^Pathologic tumor stage is according to AJCC staging for SKCM (8th edition)

### RNA isolation and qRT-PCR

Total RNA was extracted from all fresh-frozen samples using TRIzol reagent (Invitrogen, USA). The OD value (260/280) of all RNA extracted samples was greater than 1.8. For each replicate, complementary DNA (cDNA) was synthesized from 2 μg RNA using the GoScript Reverse Transcription System (Promega, USA). The qRT-PCR comprised 10 μl of GoTaq qPCR Master Mix (2×) (Promega, USA), 2 μl of diluted cDNA template (1:10) and 10 μM of each primer contributing to a total volume of 20 μl. Reactions were run in an ABI 7500 real-time PCR system (Applied Biosystems, USA) under the following conditions: 95 °C for 10 mins and 40 cycles of 95 °C for 15 s and 60 °C for 60 s. Melting curves were derived for every reaction to ensure a single product. Relative gene expression was evaluated according to the ddCT method, using the human GAPDH gene as an endogenous control for RNA load and gene expression in the analysis. All experiments were performed in triplicate. GraphPad Prism 8 (GraphPad Software, USA) was used to output figures.

The primers were as follows: *MALAT1* Fw.: GACGAGTTGTGCTGCGAT; *MALAT1* Rev.: TTCTGTGTTATGCCTGGTTA; *LINC00943* Fw.: GGATTGGATTGTGGATTGC; *LINC00943* Rev.: CAGGTCTCAGTTCAGTGTT; *LINC00261* Fw.: CTTCTTGACCACATCTTACAC; *LINC00261* Rev.: GGACCATTGCCTCTTGATT; *GAPDH* Fw: GAGAGGGAAATCGTGCGTGAC; *GAPDH* Rev.: CATCTGCTGGAAGGTGGACA.

### Multivariate cox regression model for survival analysis

To carry out a multivariate Cox regression analysis for survival analysis of patients with *MALAT1, LINC00943, and LINC00261* CNV-deficient cutaneous melanoma, we first used the UCSC genome browser (http://genome.ucsc.edu/index.html) to det*ermine the number and region of exons of MALAT1, LINC00943, and LINC00261*. All information belongs to the hg19 database (Table 2). A total of 537 SKCM patients were from the Skin Cutaneous Melanoma (TCGA, PanCancer Atlas, https://gdc.cancer.gov/about-data/publications/pancanatlas) [35] and Metastatic Melanoma (DFCI, Science 2015, https://www.ncbi.nlm.nih.gov/projects/gap/cgi-bin/study.cgi?study_id=phs000452.v2.p1) [36–38] datasets. Raw data were downloaded from cBioPortal (http://www.cbioportal.org/) [39]. By further analysing the copy number variation (CNV) data of these 537 patients, we determined whether each melanoma sample had deletions of these exons. Seg. means ≤ − 0.3 were considered CNV deficiency, others were considered without CNV deficiency (see https://docs.gdc.cancer.gov/Data/Bioinformatics_Pipelines/CNV_Pipeline/, and CNV and patient information are shown in Supplementary Table 1).

**Table 2:** Exon locus of *MALAT1, LINC00943 and LINC00261*

Gene	Exon number	Locus^a^
*MALAT1*	Exon 1	Chr 11:65265481–65,265,876
	Exon 2	Chr 11:65265159–65,265,336
	Exon 3	Chr 11:65266440–65,271,376
	Exon 4	Chr 11:65273731–65,273,902
*LINC00943*	Exon 1	Chr 12:127221553–127,221,702
	Exon 2	Chr 12:127227286–127,228,026
	Exon 3	Chr 12:127229316–127,229,434
	Exon 4	Chr 12:127229552–127,230,800
*LINC00261*	Exon 1	Chr 20:22559148–22,559,280
	Exon 2	Chr 20:22548432–22,548,523
	Exon 3	Chr 20:22547321–22,547,443
	Exon 4	Chr 20:22541192–22,545,754

^a^ The information of exons belongs to the hg19 database

To determine which factors should be included in the multivariate Cox regression model, we first performed the univariate Cox regression model for survival analysis. Factors that were statistically significant (*p* <  0.05) in the univariate Cox regression model were included in the multivariate Cox regression model, and the multivariate Cox regression model for survival analysis was performed. SPSS 22.0 was used for the analysis of the Cox regression model.

## Results

### Identification of DEMs, DELs and DEMis and reconstruction of the lncRNA-miRNA-mRNA (ceRNA) network

**Fig. 2:**
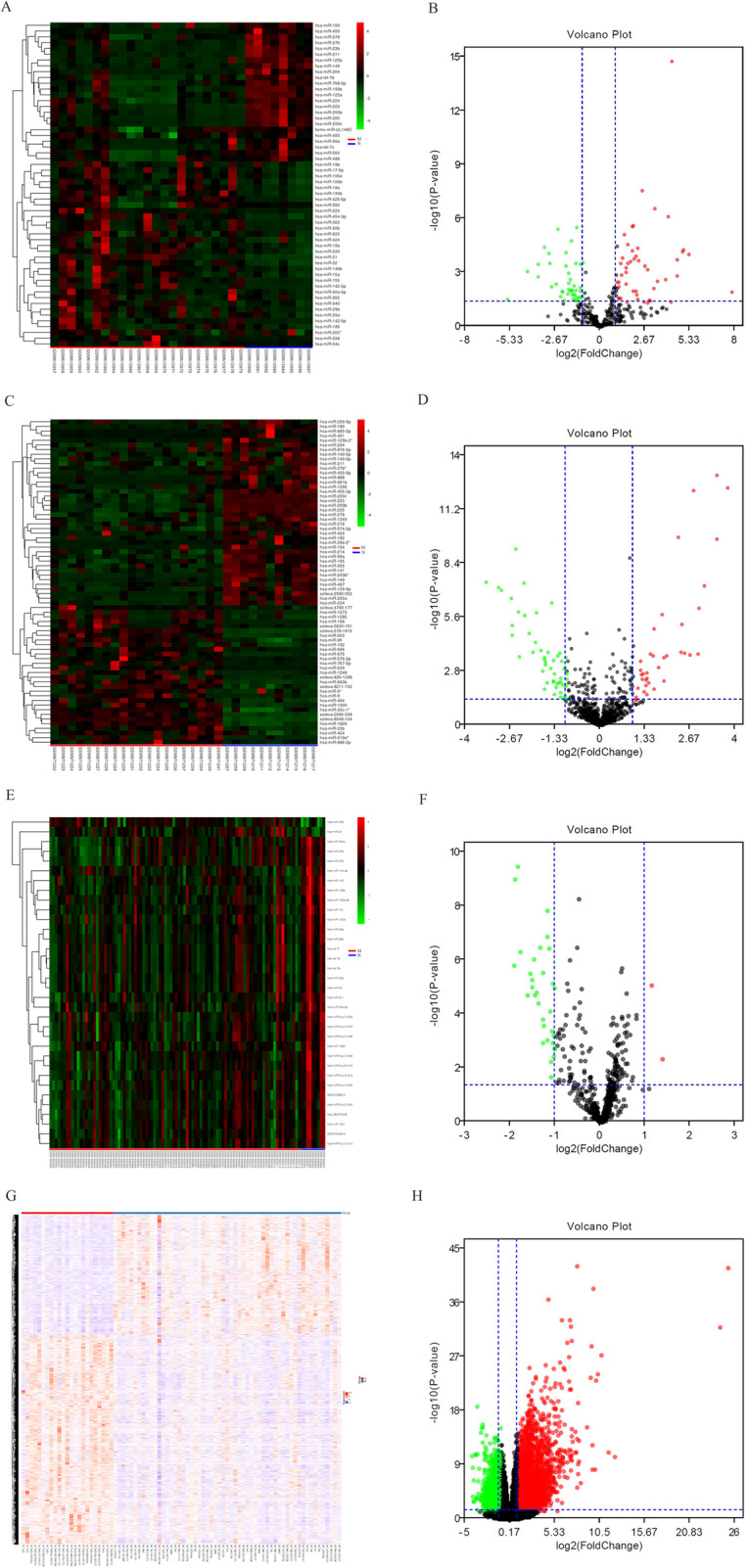
**a** Heatmap analysis of miRNA differential expressed profiles in GSE24996; (**b**) Volcano analysis of miRNA expressed profiles in GSE24996; (**c**) Heatmap analysis of miRNA differential expressed profiles in GSE35579; (**d**) Volcano analysis of miRNA expressed profiles in GSE35579; (**e**) Heatmap analysis of miRNA differential expressed profiles in GSE62372; (**f**) Volcano analysis of miRNA expressed profiles in GSE62372; (**g**) Heatmap analysis of RNA differential expressed profiles in GSE112509; (**h**) Volcano analysis of RNA expressed profiles in GSE112509. (These images were produced by R version 3.4.2)

**Fig. 3:**
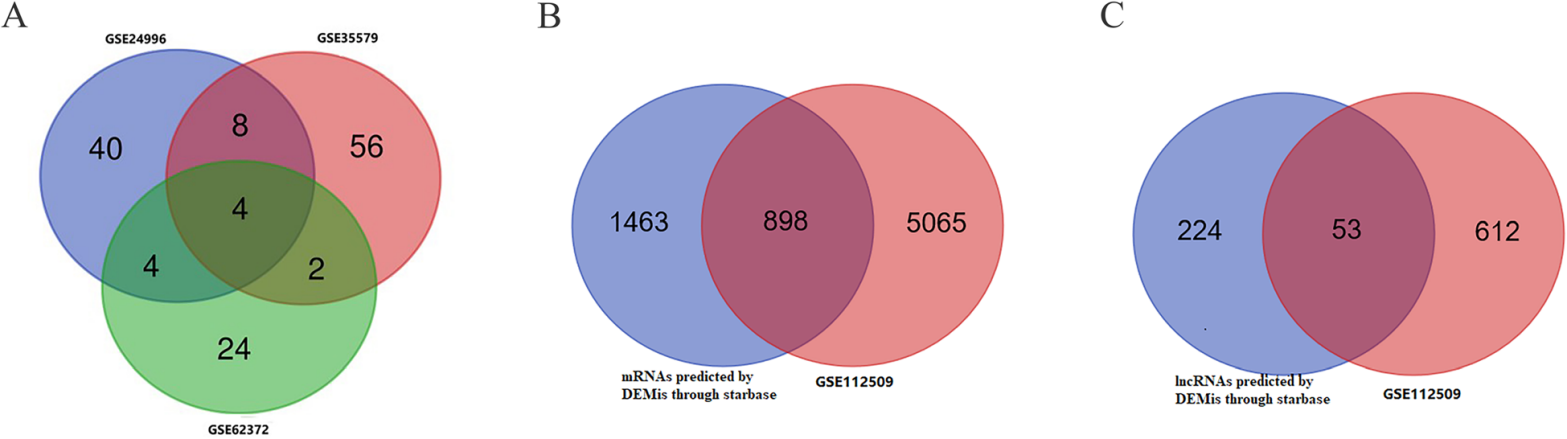
Venn diagram: (**a**) DEMis were selected with |log2FC| > 1 and adjusted *P*-value < 0.05 among the non-coding RNA profiling sets, GSE24996, GSE35579 and GSE62372. The candidates 18 miRNAs were shared in at least two datasets. **b** DEMs were selected by intersecting mRNAs predicted by DEMis through starbase and differential expressed mRNAs in GSE112509. **c** DELs were selected by intersecting lncRNAs predicted by DEMis through starbase and differential expressed lncRNAs in GSE112509. (These images were produced by R version 3.4.2)

After standardization of the GEO datasets, 56, 70 and 34 DEMis between benign nevus tissues and primary melanoma tissues were identified in GSE24996, GSE35579 and GSE62372, respectively (Supplementary Table 2, Fig. 2a-f). The candidate 18 miRNAs were shared in at least two datasets (Fig. 3a): hsa-miRNA-378a-3p, hsa-miRNA-23b-3p, hsa-miRNA-140-3p, hsa-miRNA-99a-5p, hsa-miRNA-100-5p, hsa-miRNA-204-5p, hsa-miRNA-211-5p, hsa-miRNA-205-5p, hsa-miRNA-224-5p, hsa-miRNA-200b-3p, hsa-miRNA-200c-3p, hsa-miRNA-125b-5p, hsa-miRNA-149-5p, hsa-miRNA-21-5p, hsa-miRNA-20b-5p, hsa-miRNA-424-5p, hsa-miRNA-203a-3p and hsa-miRNA-1826. According to method 2.3, 2361 mRNAs and 277 lncRNAs were predicted using these miRNAs. We ruled out two of these 18 DEMis, hsa-miRNA-203a-3p and hsa-miRNA-1826, because no predicted gene was found in starBase according to method 2.3. In addition, 5953 DEMs and 665 DELs between benign nevus tissues and primary melanoma tissues were identified in GSE112509 (Fig. 2g and h). As a result, a total of 898 DEMs and 53 DELs were selected for further analysis according to method 2.3 (Fig. 3b and c). Finally, 898 DEMs, 53 DELs and 16 DEMis were selected for further reconstruction of the lncRNA-miRNA-mRNA (ceRNA) network.

**Fig. 4:**
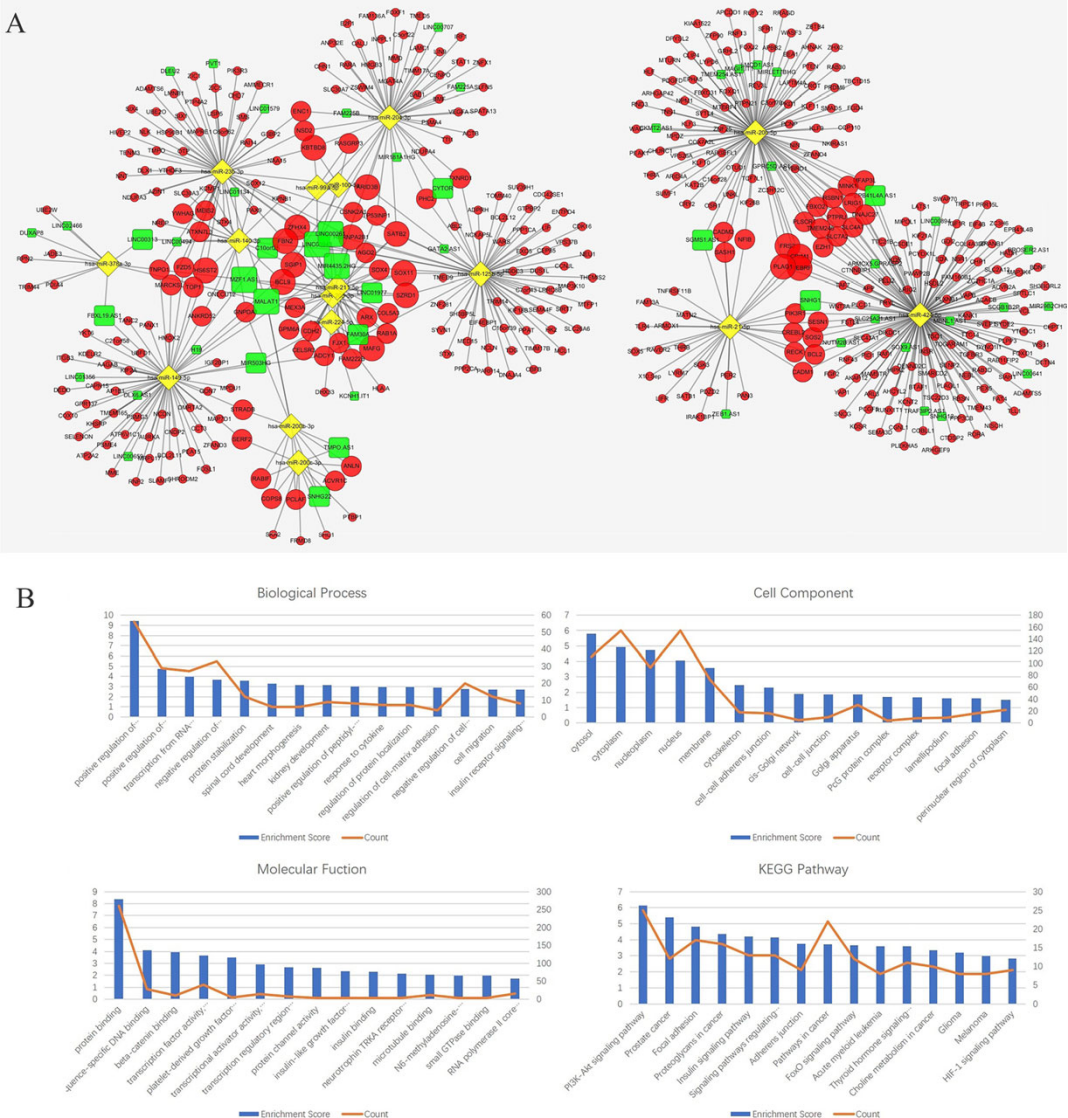
**a** ceRNA network. The round rectangle represents lncRNAs, the diamond represents miRNAs, and the ellipse represents mRNAs. There are 53 lncRNA nodes, 16 miRNA nodes, 898 mRNA nodes and 609 edges in the network. **b**-**e** Biological function and pathway analysis of differentially expressed mRNAs. **b** The top 15 significant changes in GO-BP. **c** The top 15 significant changes in the GO-CC. **d** The top 15 significant changes in the GO-MF. **e** The top 15 significant changes in the KEGG pathway. Note: more details are shown in Table 3. (Fig. 4a was produced by Cytoscape version 3.7.1)

The lncRNA-miRNA-mRNA (ceRNA) network, consisting of 53 lncRNA nodes, 16 miRNA nodes, 898 mRNA nodes and 609 edges, was reconstructed and visualized using Cytoscape (Fig. 4a).

### KEGG pathway and GO enrichment analysis of lncRNAs based on the ceRNA network

We used DAVID to analyse the biological classification of DEMs according to method 2.5. The results of the top 15 significant GO terms and KEGG pathways are shown in Table 3 and Fig. 4b-e. Sixty pathways were significantly enriched through KEGG pathway analysis, including the PI3K-Akt signalling pathway, focal adhesion, proteoglycans in cancer, pathway in cancer and, most importantly, melanomagenesis. The results of GO-BP analysis revealed 172 enriched terms, particularly in the regulation of transcription, such as positive regulation of transcription from the RNA polymerase II promoter, positive regulation of transcription (DNA-templated), and transcription from the RNA polymerase II promoter.

**Table 3:** The top 15 significant changes in GO-BP (A), −CC (B), −MF(C) and KEGG pathway (D) according to differentially expressed genes in ceRNA network

A				
GO-BP Term	Enrichment Score	Count	%	*P*-Value
positive regulation of transcription from RNA polymerase II promoter	9.446887	56	13.18	< 0.001
positive regulation of transcription, DNA-templated	4.759462	29	6.824	< 0.001
transcription from RNA polymerase II promoter	3.957811	27	6.353	< 0.001
negative regulation of transcription from RNA polymerase II promoter	3.674737	33	7.765	< 0.001
protein stabilization	3.580807	12	2.824	< 0.001
spinal cord development	3.291952	6	1.412	< 0.001
heart morphogenesis	3.157839	6	1.412	< 0.001
kidney development	3.144958	9	2.118	< 0.001
positive regulation of peptidyl-serine phosphorylation	3.001168	8	1.882	< 0.001
response to cytokine	2.967806	7	1.647	0.001
regulation of protein localization	2.967806	7	1.647	0.001
regulation of cell-matrix adhesion	2.914902	4	0.941	0.001
negative regulation of cell proliferation	2.759652	20	4.706	0.002
cell migration	2.732195	12	2.824	0.002
insulin receptor signaling pathway	2.724648	8	1.882	0.002
B				
GO-CC Term	Enrichment Score	Count	%	*P*-Value
cytosol	5.793638	111	26.12	< 0.001
cytoplasm	4.942099	154	36.24	< 0.001
nucleoplasm	4.725908	93	21.88	< 0.001
nucleus	4.05725	154	36.24	< 0.001
membrane	3.599508	73	17.18	< 0.001
cytoskeleton	2.478053	18	4.235	0.003
cell-cell adherens junction	2.302618	16	3.765	0.005
cis-Golgi network	1.888299	5	1.176	0.013
cell-cell junction	1.877361	10	2.353	0.013
Golgi apparatus	1.852153	30	7.059	0.014
PcG protein complex	1.690927	4	0.941	0.02
receptor complex	1.672147	8	1.882	0.021
lamellipodium	1.616858	9	2.118	0.024
focal adhesion	1.603246	16	3.765	0.025
perinuclear region of cytoplasm	1.496331	22	5.176	0.032
C				
GO-MF Term	Enrichment Score	Count	%	*P*-Value
protein binding	8.364509	260	61.18	< 0.001
sequence-specific DNA binding	4.118515	28	6.588	< 0.001
beta-catenin binding	3.946374	10	2.353	< 0.001
transcription factor activity, sequence-specific DNA binding	3.635935	41	9.647	< 0.001
platelet-derived growth factor receptor binding	3.50464	5	1.176	< 0.001
transcriptional activator activity, RNA polymerase II core promoter proximal region sequence-specific binding	2.912949	15	3.529	0.001
transcription regulatory region sequence-specific DNA binding	2.667561	7	1.647	0.002
protein channel activity	2.637341	4	0.941	0.002
insulin-like growth factor receptor binding	2.344093	4	0.941	0.005
insulin binding	2.293839	3	0.706	0.005
neurotrophin TRKA receptor binding	2.124416	3	0.706	0.008
microtubule binding	2.037592	12	2.824	0.009
N6-methyladenosine-containing RNA binding	1.984943	3	0.706	0.01
small GTPase binding	1.982255	4	0.941	0.01
RNA polymerase II core promoter proximal region sequence-specific DNA binding	1.726919	16	3.765	0.019
D				
KEGG pathway	Enrichment Score	Count	%	*P*-Value
PI3K-Akt signaling pathway	6.144606	25	5.882	< 0.001
Prostate cancer	5.389517	12	2.824	< 0.001
Focal adhesion	4.815445	17	4	< 0.001
Proteoglycans in cancer	4.365137	16	3.765	< 0.001
Insulin signaling pathway	4.202316	13	3.059	< 0.001
Signaling pathways regulating pluripotency of stem cells	4.141148	13	3.059	< 0.001
Adherens junction	3.732503	9	2.118	< 0.001
Pathways in cancer	3.709619	22	5.176	< 0.001
FoxO signaling pathway	3.670169	12	2.824	< 0.001
Acute myeloid leukemia	3.609095	8	1.882	< 0.001
Thyroid hormone signaling pathway	3.584028	11	2.588	< 0.001
Choline metabolism in cancer	3.353402	10	2.353	< 0.001
Glioma	3.20572	8	1.882	< 0.001
Melanoma	2.973883	8	1.882	0.001
HIF-1 signaling pathway	2.844366	9	2.118	0.001

### Hub gene selection

According to the node degree in the ceRNA network, we found that three lncRNAs, *MALAT1, LINC00943, and LINC00261*, had the highest number of lncRNA-miRNA and miRNA-mRNA pairs, suggesting that these three lncRNAs could be chosen as hub nodes, and the results are shown in Table 4. Therefore, these three lncRNAs might play an essential role in melanomagenesis and might be considered key lncRNAs.

**Table 4:** The number of the highest lncRNA–miRNA and miRNA–mRNA pairs

	lncRNA-miRNA pairs	miRNA-mRNA pairs	Total number
MALAT1	9	200	209
LINC00943	7	202	209
LINC00261	5	158	163

### Reconstruction of the *MALAT1/LINC00943/LINC00261*-miRNA-mRNA subnetworks

**Fig. 5:**
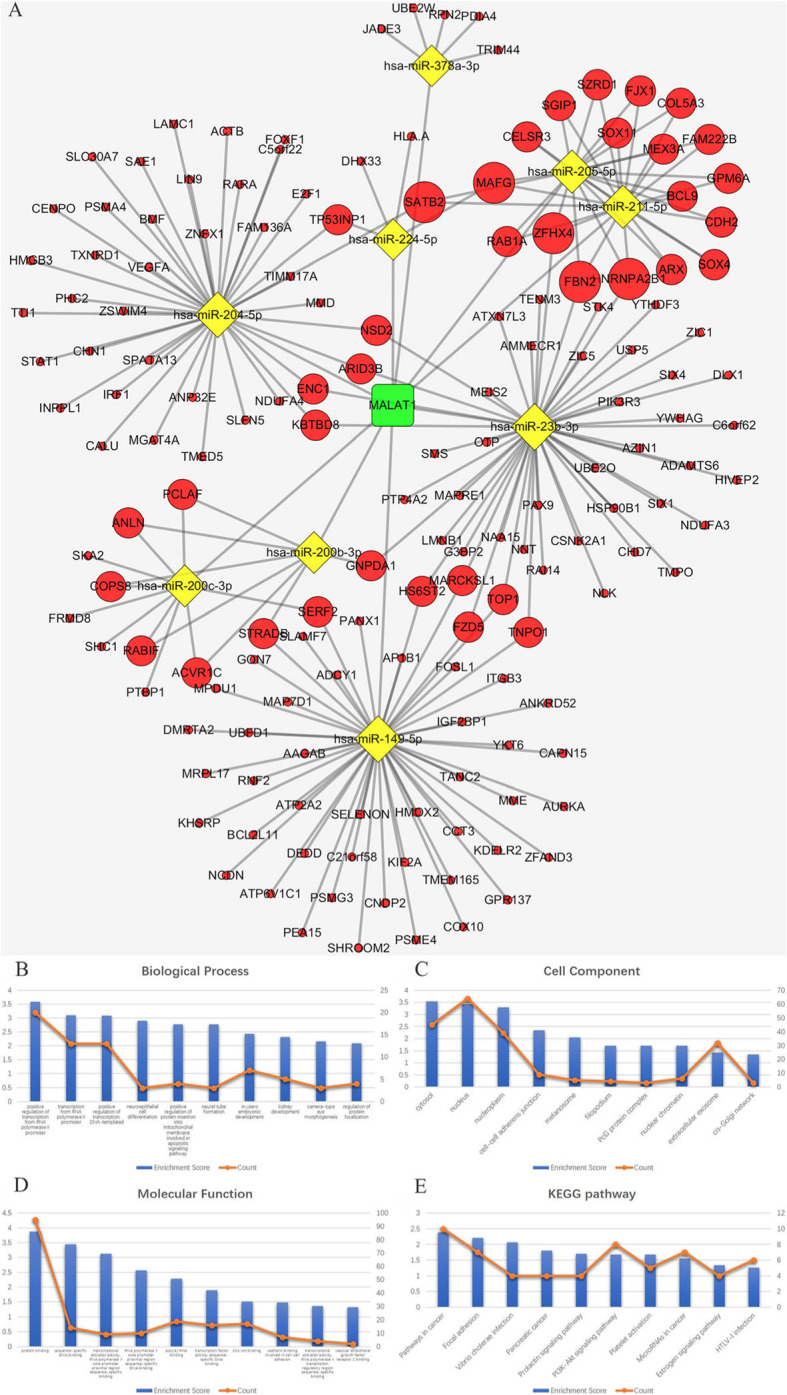
**a** The ceRNA sub-network of *MALAT1*. The round rectangle represents lncRNAs, the diamond represents miRNAs, and the ellipse represents mRNAs. There are 1 lncRNA nodes, 9 miRNA nodes, 158 mRNA nodes and 209 edges in the network. **b**-**e** Biological function and pathway analysis of *MALAT1* paired mRNAs. **b** The top 10 significant changes in the GO-BP. **c** The top 10 significant changes in the GO-CC. **d** The top 10 significant changes in the GO-MF. **e** The top 10 significant changes in the KEGG pathway. Note: more details are shown in Table 5. (Fig. 5a was produced by Cytoscape version 3.7.1)

**Fig. 6:**
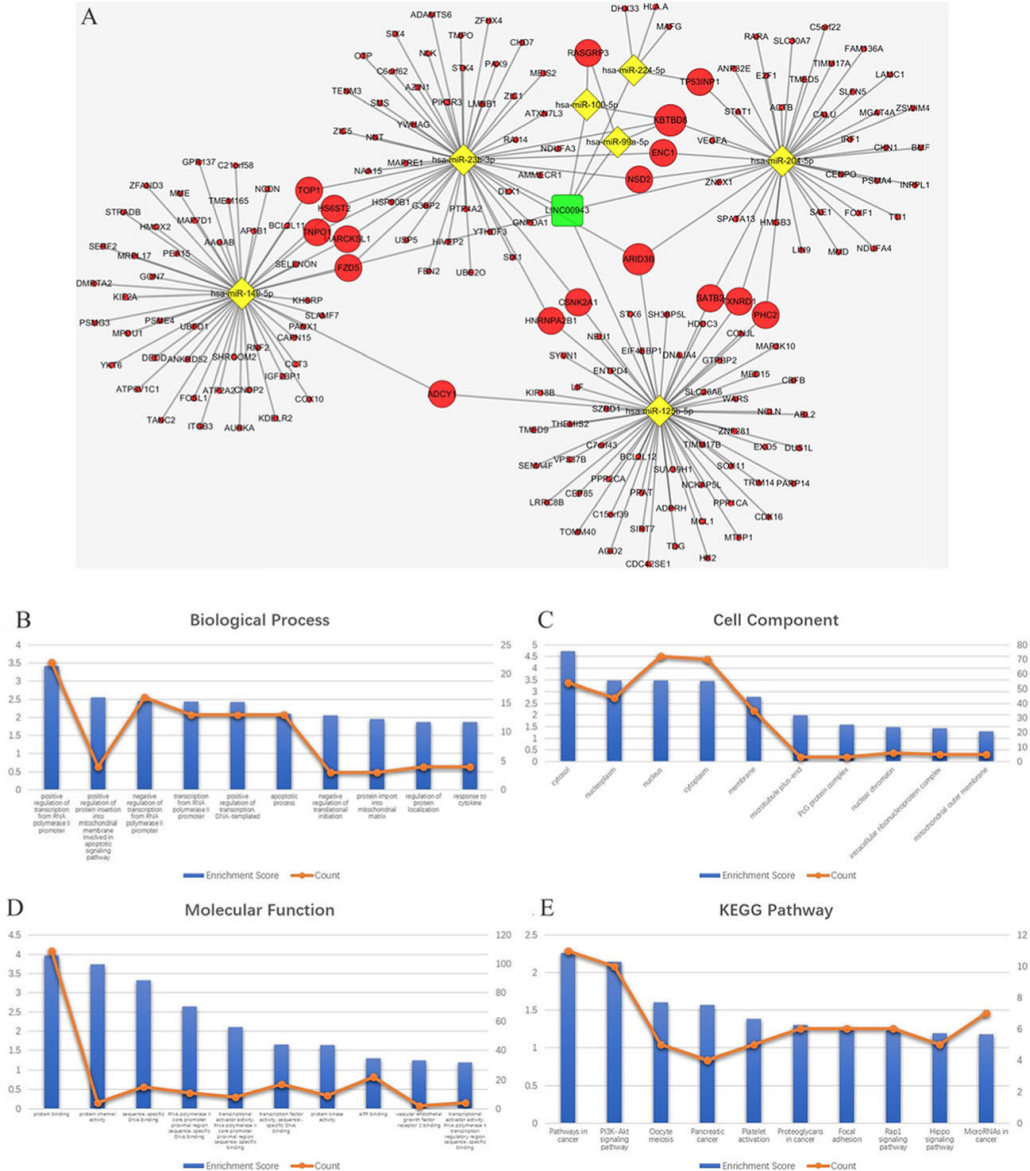
**a** The ceRNA sub-network of *LINC00943*. The round rectangle represents lncRNAs, the diamond represents miRNAs, and the ellipse represents mRNAs. There are 1 lncRNA nodes, 7 miRNA nodes, 182 mRNA nodes and 209 edges in the network. **b**-**e** Biological function and pathway analysis of *LINC00943* paired mRNAs. **b** The top 10 significant changes in the GO-BP. **c** The top 10 significant changes in the GO-CC. **d** The top 10 significant changes in the GO-MF. **e** The top 10 significant changes in the KEGG pathway. Note: more details are shown in Table 6. (Fig. 6a was generated by Cytoscape version 3.7.1)

**Fig. 7:**
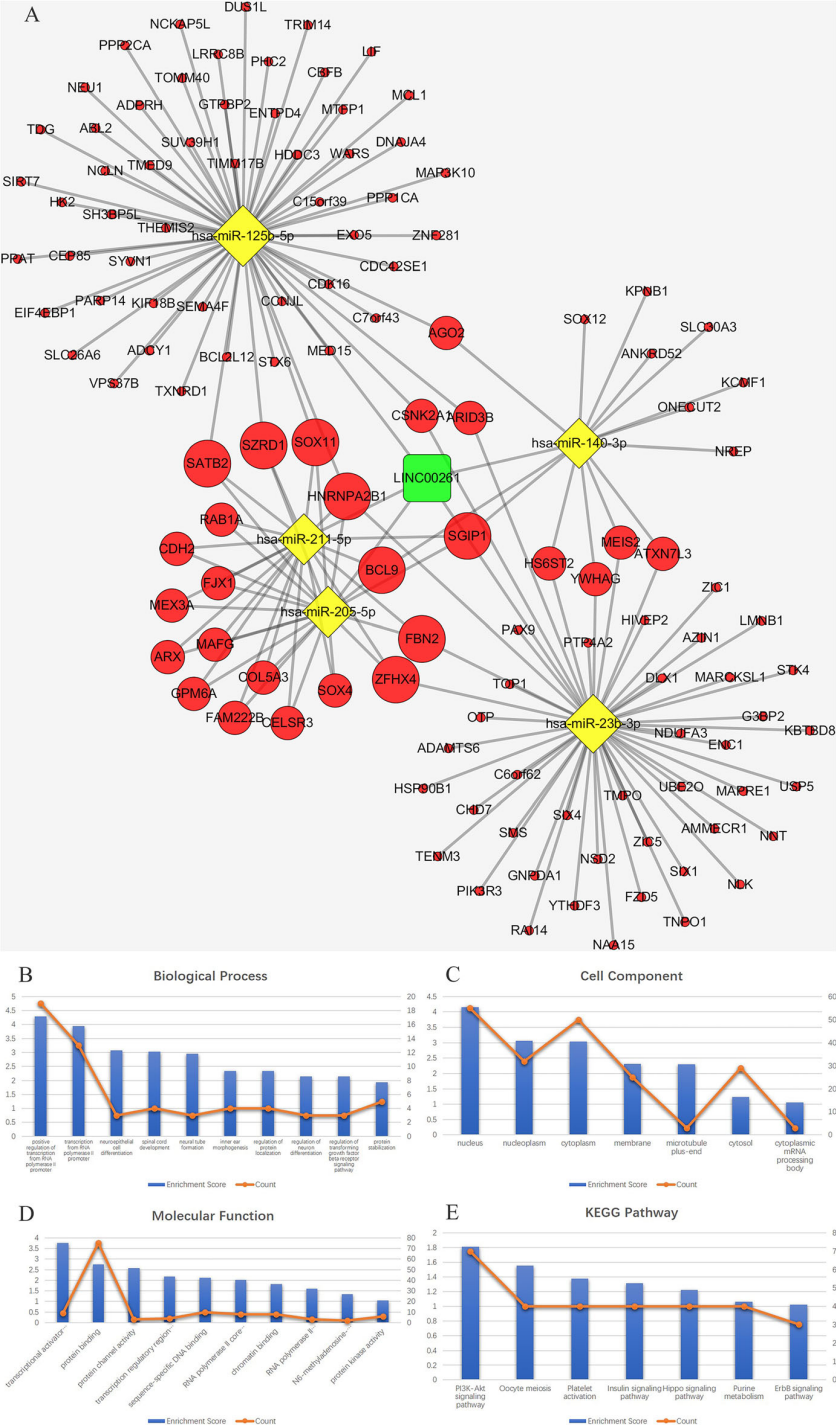
**a** The ceRNA sub-network of *LINC00261*. The round rectangle represents lncRNAs, the diamond represents miRNAs, and the ellipse represents mRNAs. There are 1 lncRNA nodes, 5 miRNA nodes, 123 mRNA nodes and 163 edges in the network. **b**-**e** Biological function and pathway analysis of *LINC00261* paired mRNAs. **b** The top 10 significant changes in the GO-BP. **c** The changes in the GO-CC. **d** The top 10 significant changes in the GO-MF. **e** The changes in the KEGG pathway. Note: more details are shown in Table 7. (Fig. 7a was generated by Cytoscape version 3.7.1)

*MALAT, LINC00943, LINC00261* and their paired miRNAs and mRNAs were used to reconstruct key ceRNA subnetworks. The *MALAT1* ceRNA network consists of 1 lncRNA node, 9 miRNA nodes, 158 mRNA nodes and 209 edges, as shown in Fig. 5a. The *LINC00943* ceRNA network consists of 1 lncRNA node, 7 miRNA nodes, 182 mRNA nodes and 209 edges, as shown in Fig. 6a. The *LINC00261* ceRNA network consists of 1 lncRNA node, 5 miRNA nodes, 123 mRNA nodes and 163 edges, as shown in Fig. 7a. The results of functional analysis revealed that 75 GO-BP, 21 GO-CC, 15 GO-MF and 20 pathways were enriched in the *MALAT1*-miRNA-mRNA subnetwork; 67 GO-BP, 14 GO-CC, 17 GO-MF and 13 pathways were enriched in the *LINC00943*-miRNA-mRNA subnetwork; and 42 GO-BP, 7 GO-CC, 10 GO-MF and 7 pathways were enriched in the *LINC00261*-miRNA-mRNA subnetwork. The results of the top 10 significant GO terms and KEGG pathways of these three lncRNAs are shown in Fig. 5b-e, Fig. 6b-e, Fig. 7b-e, and Tables 5, 6, 7.

**Table 5:** The top 15 significant changes in GO-BP (A), −CC (B), −MF(C) and KEGG pathway (D) according to differentially expressed genes in *MALAT1-*ceRNA sub-network

A				
GO-BP Term	Enrichment Score	Count	%	*P*-Value
positive regulation of transcription from RNA polymerase II promoter	3.579259	20	11.43	< 0.001
transcription from RNA polymerase II promoter	3.106442	13	7.429	< 0.001
positive regulation of transcription, DNA-templated	3.091753	13	7.429	< 0.001
neuroepithelial cell differentiation	2.894845	3	1.714	0.001
positive regulation of protein insertion into mitochondrial membrane involved in apoptotic signaling pathway	2.772993	4	2.286	0.002
neural tube formation	2.772164	3	1.714	0.002
in utero embryonic development	2.425229	7	4	0.004
kidney development	2.315189	5	2.857	0.005
camera-type eye morphogenesis	2.158154	3	1.714	0.007
regulation of protein localization	2.092037	4	2.286	0.008
inner ear morphogenesis	2.092037	4	2.286	0.008
positive regulation of branching involved in ureteric bud morphogenesis	2.011071	3	1.714	0.01
positive regulation of neuroblast proliferation	1.967555	3	1.714	0.011
negative regulation of transcription from RNA polymerase II promoter	1.923719	13	7.429	0.012
cell migration	1.922663	6	3.429	0.012
B				
GO-CC Term	Enrichment Score	Count	%	*P*-Value
cytosol	3.530641	45	25.71	< 0.001
nucleus	3.429028	64	36.57	< 0.001
nucleoplasm	3.288165	39	22.29	< 0.001
cell-cell adherens junction	2.341584	9	5.143	0.005
melanosome	2.052614	5	2.857	0.009
filopodium	1.71293	4	2.286	0.019
PcG protein complex	1.707154	3	1.714	0.02
nuclear chromatin	1.705842	6	3.429	0.02
extracellular exosome	1.429256	32	18.29	0.037
cis-Golgi network	1.35117	3	1.714	0.045
spindle microtubule	1.314751	3	1.714	0.048
cytoplasm	1.239444	52	29.71	0.058
perinuclear region of cytoplasm	1.205186	10	5.714	0.062
membrane	1.146558	25	14.29	0.071
spindle	1.134303	4	2.286	0.073
C				
GO-MF Term	Enrichment Score	Count	%	*P*-Value
protein binding	3.880727	95	54.29	< 0.001
sequence-specific DNA binding	3.451663	14	8	< 0.001
transcriptional activator activity, RNA polymerase II core promoter proximal region sequence-specific binding	3.120112	9	5.143	< 0.001
RNA polymerase II core promoter proximal region sequence-specific DNA binding	2.566023	10	5.714	0.003
poly(A) RNA binding	2.27862	19	10.86	0.005
transcription factor activity, sequence-specific DNA binding	1.893028	16	9.143	0.013
zinc ion binding	1.508313	17	9.714	0.031
cadherin binding involved in cell-cell adhesion	1.481723	7	4	0.033
transcriptional activator activity, RNA polymerase II transcription regulatory region sequence-specific binding	1.359345	4	2.286	0.044
vascular endothelial growth factor receptor 2 binding	1.315103	2	1.143	0.048
N6-methyladenosine-containing RNA binding	1.249923	2	1.143	0.056
mRNA 5′-UTR binding	1.144306	2	1.143	0.072
protein heterodimerization activity	1.046398	8	4.571	0.09
RNA polymerase II regulatory region sequence-specific DNA binding	1.031152	5	2.857	0.093
DNA binding	1.029587	20	11.43	0.093
D				
GO-MF Term	Enrichment Score	Count	%	*P*-Value
protein binding	3.880727	95	54.29	< 0.001
sequence-specific DNA binding	3.451663	14	8	< 0.001
transcriptional activator activity, RNA polymerase II core promoter proximal region sequence-specific binding	3.120112	9	5.143	< 0.001
RNA polymerase II core promoter proximal region sequence-specific DNA binding	2.566023	10	5.714	0.003
poly(A) RNA binding	2.27862	19	10.86	0.005
transcription factor activity, sequence-specific DNA binding	1.893028	16	9.143	0.013
zinc ion binding	1.508313	17	9.714	0.031
cadherin binding involved in cell-cell adhesion	1.481723	7	4	0.033
transcriptional activator activity, RNA polymerase II transcription regulatory region sequence-specific binding	1.359345	4	2.286	0.044
vascular endothelial growth factor receptor 2 binding	1.315103	2	1.143	0.048
N6-methyladenosine-containing RNA binding	1.249923	2	1.143	0.056
mRNA 5′-UTR binding	1.144306	2	1.143	0.072
protein heterodimerization activity	1.046398	8	4.571	0.09
RNA polymerase II regulatory region sequence-specific DNA binding	1.031152	5	2.857	0.093
DNA binding	1.029587	20	11.43	0.093

**Table 6:** The top significant changes in GO-BP (A), −CC (B), −MF(C) and KEGG pathway (D) according to differentially expressed genes in *LINC00943-*ceRNA sub-network

A				
GO-BP Term	Enrichment Score	Count	%	*P*-Value
positive regulation of transcription from RNA polymerase II promoter	3.413985	22	12.22	< 0.001
positive regulation of protein insertion into mitochondrial membrane involved in apoptotic signaling pathway	2.5522952	4	2.222	0.003
negative regulation of transcription from RNA polymerase II promoter	2.4555568	16	8.889	0.004
transcription from RNA polymerase II promoter	2.4471842	13	7.222	0.004
positive regulation of transcription, DNA-templated	2.4336944	13	7.222	0.004
apoptotic process	2.1092412	13	7.222	0.008
negative regulation of translational initiation	2.064083	3	1.667	0.009
protein import into mitochondrial matrix	1.95711	3	1.667	0.011
regulation of protein localization	1.8821494	4	2.222	0.013
response to cytokine	1.8821494	4	2.222	0.013
cellular response to cytokine stimulus	1.7404426	3	1.667	0.018
cell morphogenesis	1.6784701	4	2.222	0.021
positive regulation of mesenchymal cell proliferation	1.6028585	3	1.667	0.025
intracellular protein transport	1.6019839	7	3.889	0.025
protein sumoylation	1.5991972	5	2.778	0.025
B				
GO-CC Term	Enrichment Score	Count	%	*P*-Value
cytosol	4.721026	54	30	< 0.001
nucleoplasm	3.468485	44	24.44	< 0.001
nucleus	3.459493	72	40	< 0.001
cytoplasm	3.448156	70	38.89	< 0.001
membrane	2.786622	35	19.44	0.002
microtubule plus-end	1.979181	3	1.667	0.01
PcG protein complex	1.593489	3	1.667	0.025
nuclear chromatin	1.476598	6	3.333	0.033
intracellular ribonucleoprotein complex	1.428852	5	2.778	0.037
mitochondrial outer membrane	1.3075	5	2.778	0.049
endoplasmic reticulum membrane	1.253057	14	7.778	0.056
perinuclear region of cytoplasm	1.207393	11	6.111	0.062
MLL5-L complex	1.146143	2	1.111	0.071
mitochondrial inner membrane presequence translocase complex	1.096965	2	1.111	0.08
C				
GO-MF Term	Enrichment Score	Count	%	*P*-Value
protein binding	3.972219	109	60.56	< 0.001
protein channel activity	3.7469	4	2.222	< 0.001
sequence-specific DNA binding	3.320627	15	8.333	< 0.001
RNA polymerase II core promoter proximal region sequence-specific DNA binding	2.640286	11	6.111	0.002
transcriptional activator activity, RNA polymerase II core promoter proximal region sequence-specific binding	2.106865	8	4.444	0.008
transcription factor activity, sequence-specific DNA binding	1.648871	17	9.444	0.022
protein kinase activity	1.643895	9	5	0.023
ATP binding	1.307149	22	12.22	0.049
vascular endothelial growth factor receptor 2 binding	1.25008	2	1.111	0.056
transcriptional activator activity, RNA polymerase II transcription regulatory region sequence-specific binding	1.197916	4	2.222	0.063
N6-methyladenosine-containing RNA binding	1.185193	2	1.111	0.065
P-P-bond-hydrolysis-driven protein transmembrane transporter activity	1.129258	2	1.111	0.074
poly(A) RNA binding	1.119963	17	9.444	0.076
chromatin binding	1.08038	8	4.444	0.083
mRNA 5′-UTR binding	1.080159	2	1.111	0.083
D				
KEGG pathway	Enrichment Score	Count	%	*P*-Value
Pathways in cancer	2.26453	11	6.111	0.005
PI3K-Akt signaling pathway	2.145933	10	5.556	0.007
Oocyte meiosis	1.604046	5	2.778	0.025
Pancreatic cancer	1.566902	4	2.222	0.027
Platelet activation	1.386975	5	2.778	0.041
Insulin signaling pathway	1.307592	5	2.778	0.049
Proteoglycans in cancer	1.304184	6	3.333	0.05
Focal adhesion	1.259046	6	3.333	0.055
Rap1 signaling pathway	1.229991	6	3.333	0.059
Hippo signaling pathway	1.190921	5	2.778	0.064
MicroRNAs in cancer	1.179653	7	3.889	0.066
HIF-1 signaling pathway	1.146419	4	2.222	0.071
Vibrio cholerae infection	1.020041	3	1.667	0.095

**Table 7:** The top significant changes in GO-BP (A), −CC (B), −MF(C) and KEGG pathway (D) according to differentially expressed genes in *LINC00261-*ceRNA sub-network

A				
GO-BP Term	Enrichment Score	Count	%	*P*-Value
positive regulation of transcription from RNA polymerase II promoter	4.294676	19	14.29	< 0.001
transcription from RNA polymerase II promoter	3.946596	13	9.774	< 0.001
neuroepithelial cell differentiation	3.074302	3	2.256	< 0.001
spinal cord development	3.033527	4	3.008	0.001
neural tube formation	2.951191	3	2.256	0.001
inner ear morphogenesis	2.342119	4	3.008	0.005
regulation of protein localization	2.342119	4	3.008	0.005
regulation of neuron differentiation	2.141452	3	2.256	0.007
regulation of transforming growth factor beta receptor signaling pathway	2.141452	3	2.256	0.007
protein stabilization	1.937969	5	3.759	0.012
fungiform papilla morphogenesis	1.892035	2	1.504	0.013
stem cell differentiation	1.887589	3	2.256	0.013
regulation of signal transduction	1.799832	3	2.256	0.016
negative regulation of transcription from RNA polymerase II promoter	1.756023	11	8.271	0.018
myotome development	1.717328	2	1.504	0.019
B				
GO-CC Term	Enrichment Score	Count	%	*P*-Value
nucleus	4.161906	55	41.35	< 0.001
nucleoplasm	3.062718	32	24.06	< 0.001
cytoplasm	3.032352	50	37.59	< 0.001
membrane	2.306958	25	18.8	0.005
microtubule plus-end	2.297019	3	2.256	0.005
cytosol	1.228885	29	21.8	0.059
cytoplasmic mRNA processing body	1.060323	3	2.256	0.087
C				
GO-MF Term	Enrichment Score	Count	%	*P*-Value
transcriptional activator activity, RNA polymerase II core promoter proximal region sequence-specific binding	3.752771	9	6.767	< 0.001
protein binding	2.747245	75	56.39	0.002
protein channel activity	2.559552	3	2.256	0.003
transcription regulatory region sequence-specific DNA binding	2.171926	4	3.008	0.007
sequence-specific DNA binding	2.120902	10	7.519	0.008
RNA polymerase II core promoter proximal region sequence-specific DNA binding	2.024814	8	6.015	0.009
chromatin binding	1.812398	8	6.015	0.015
RNA polymerase II transcription coactivator activity	1.602367	3	2.256	0.025
N6-methyladenosine-containing RNA binding	1.341641	2	1.504	0.046
protein kinase activity	1.042052	6	4.511	0.091
D				
KEGG pathway	Enrichment Score	Count	%	*P*-Value
PI3K-Akt signaling pathway	1.809894	7	5.263	0.015
Oocyte meiosis	1.553469	4	3.008	0.028
Platelet activation	1.379399	4	3.008	0.042
Insulin signaling pathway	1.315081	4	3.008	0.048
Hippo signaling pathway	1.219786	4	3.008	0.06
Purine metabolism	1.062637	4	3.008	0.087
ErbB signaling pathway	1.024741	3	2.256	0.094

### Expression of *MALAT1, LINC00943* and *LINC00261* is higher in tumour tissues

**Fig. 8:**
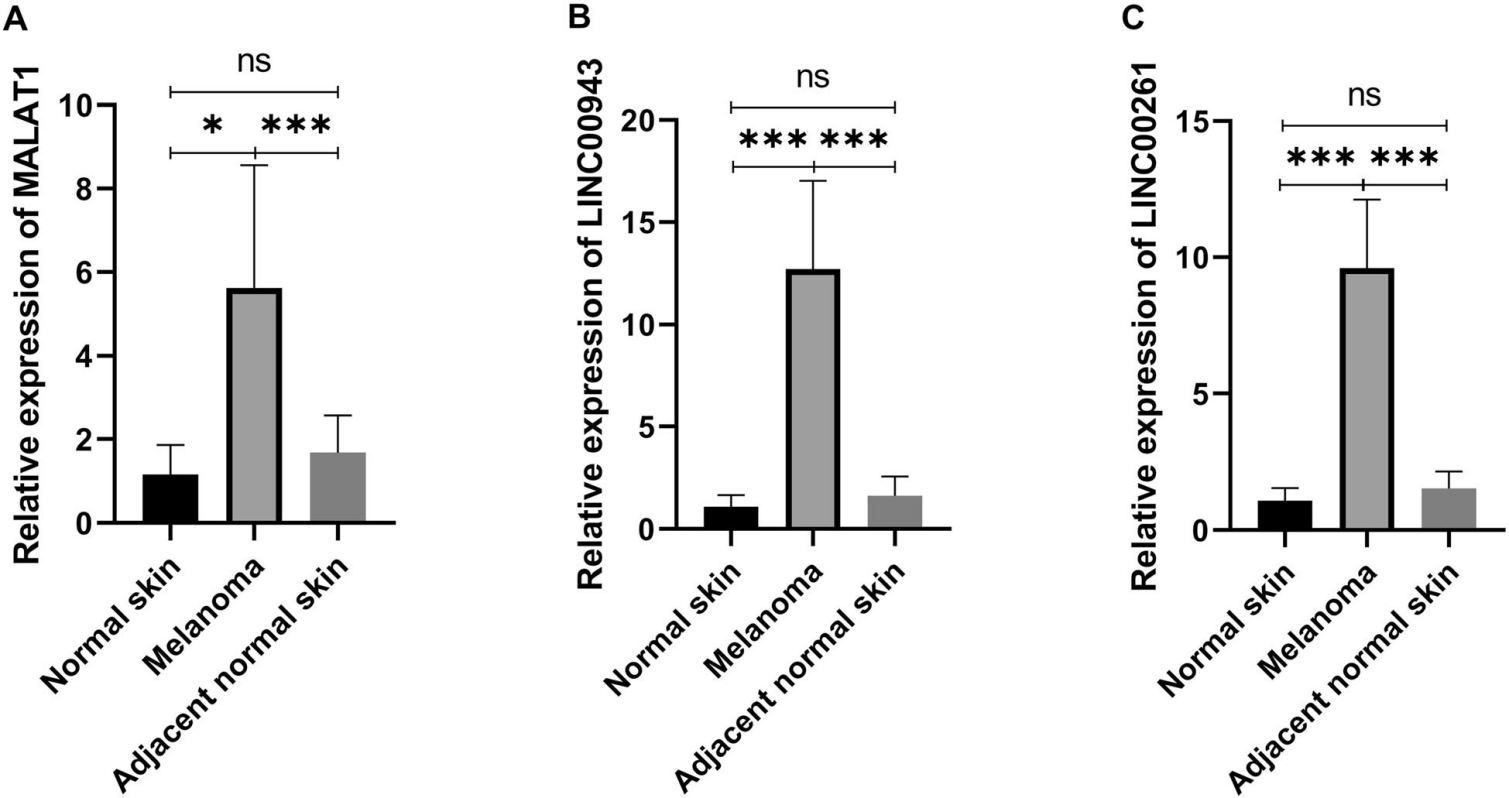
The expression level of *MALAT1* (**a**), *LINC00943* (**b**) and *LINC00261* (**c**) in normal skin, adjacent normal skin and melanoma tissues

To confirm the expression of *MALAT1, LINC00943* and *LINC00261* in melanoma tissues, we evaluated the *MALAT1, LINC00943* and *LINC00261* expression levels in the cancer tissues from 12 melanoma patients (see Table 1) and 3 healthy tissues via qRT-PCR, as shown in Fig. 8. The results showed that the expression of *MALAT1, LINC00943* and *LINC00261* was significantly higher in the tumour tissues than in the healthy tissues (*p* = 0.0243, *p* = 0.0005, *p* <  0.0001, respectively). Additionally, the expression of *MALAT1, LINC00943* and *LINC00261* was significantly higher in the tumour tissues than in the adjacent normal tissues (*p* = 0.0002, *p* <  0.0001, *p* <  0.0001, respectively). However, no significant difference was observed between the healthy tissues and the adjacent normal skin tissues in the expression of *MALAT1, LINC00943* and *LINC00261* (*p =* 0.366, *p =* 0.379, *p =* 0.262, respectively). The results are consistent with those discussed above. Thus, the expression of *MALAT1, LINC00943* and *LINC00261* is increased in melanoma and may be responsible for the tumorigenesis of melanoma.

### *MALAT1* and *LINC00943* are independent risk factors for the prognosis of cutaneous melanoma

**Fig. 9:**
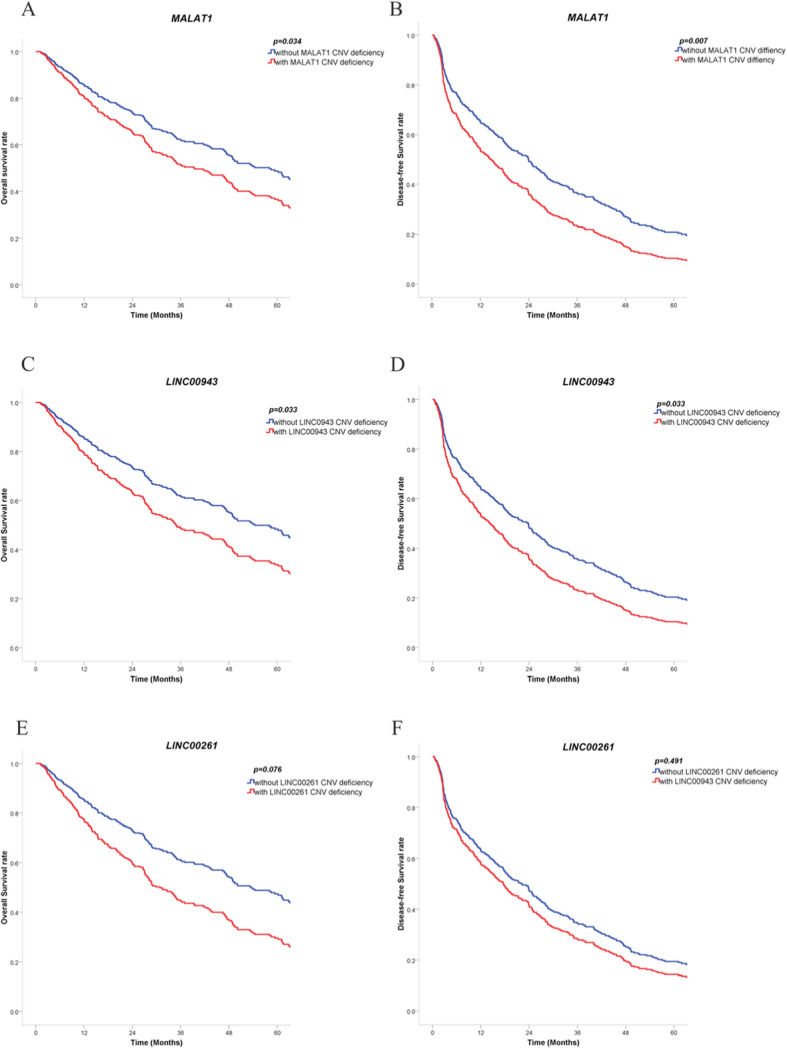
Multivariate COX regression model for survival analysis of *MALAT1* (**a**, **b**), *LINC00943* (**c**, **d**) and *LINC00261* (**e**, **f**). (This image was generated by SPSS version 22.0)

A univariate Cox regression model for survival analysis of age, sex and stage was performed, and the results are shown in Supplementary Table 3. Then, the multivariate Cox regression model for survival analysis of *MALAT1*, *LINC00943*, and *LINC00261* was performed. The results showed that the overall survival time and disease-free survival time of the patients with *MALAT1* or *LINC00943* CNV deficiency were significantly lower than those without it, and the difference was significant (details are shown in Table 8 and Fig. 9a-d), suggesting that *MALAT1* and *LINC00943* are independent risk factors for the prognosis of cutaneous melanoma. Although the overall survival time and disease-free survival time of patients with *LINC00261* deletion were lower than those without it, the difference was not significant (*p* = 0.535, *p* = 0.694) (details are shown in Table 8 and Fig. 9e- f).

**Table 8:** Multivariate COX regression model for overall survival (A) and disease-free survival analysis (B) of *MALAT1*, *LINC00943*, and *LINC00261*

A						
	Number of cases, Total	Number of cases, Decased	Median Months, Overall	OR	95%CI	*p*-value
MALAT1						
with CNV deficiency	82	53	34.23	0.714	0.524–0.975	0.034
without CNV deficiency	454	243	63.53			
LINC00943						
with CNV deficiency	54	34	55.59	0.671	0.465–0.969	0.033
without CNV deficiency	482	262	61.05			
LINC00261						
with CNV deficiency	23	16	17.03	0.612	0.356–1.053	0.076
without CNV deficiency	513	280	61.05			
B						
	Number of cases, Total	Number of cases, Decased	Median Months, Overall	OR	95%CI	*p*-value
MALAT1						
with CNV deficiency	84	69	15.52	0.691	0.528–0.906	0.007
without CNV deficiency	448	331	27.09			
LINC00943						
with CNV deficiency	55	45	21.37	0.704	0.511–0.971	0.033
without CNV deficiency	477	355	24.82			
LINC00261						
with CNV deficiency	23	19	13.50	0.842	0.516–1.374	0.491
without CNV deficiency	509	381	25.02

## Discussion

In this study, three lncRNAs, *MALAT1, LINC00943 and LINC00261,* were identified according to the reconstructed ceRNA network. Among these key lncRNAs found in this study, *MALAT1* has been demonstrated to be related to various malignant tumours [40–44]. Studies have confirmed that *MALAT1* is a valuable prognostic marker and a promising therapeutic target in lung cancer metastasis [40, 41]. A study also suggested that *MALAT1* plays an important role in tumour progression and could serve as a promising therapeutic target [42]. Through the study of the whole-genome mutational landscape and characterization of noncoding and structural mutations in liver cancer, Fujimoto A. and colleagues discovered that *MALAT1 is* closely related to liver carcinogenesis.^46^ In addition, a study revealed a novel mechanism of *MALAT1*-regulated autophagy-related chemoresistance in gastric cancer [44]. At present, it is believed that *MALAT1* is mainly responsible for regulating the proliferation, migration and invasion of tumour cells. According to our findings, *MALAT1* might also be a crucial factor in the tumorigenesis and development of melanoma. In this subnetwork, we found nine lncRNA-miRNA pairs: miRNA-378a-3p, miRNA-23b-3p, miRNA-224-5p, miRNA-204-5p, miRNA-205-5p, miRNA-200c-3p, miRNA-200b-3p, miRNA-149-5p, and miRNA-211-5p. Among them, *MALAT1* was shown to regulate chemoresistance via miRNA-23b-3p sequestration in gastric cancer [44]. In ovarian cancer, a study suggested that *MALAT1*-miRNA-211-5p may act as a key mediator in the prevention of this disease [45]. *MALAT1* is also involved in promoting renal cell carcinoma through interaction with miRNA-205-5p [46]. Studies have confirmed that *MALAT1* functions in liver and lung cancer through miRNA-204-5p [47, 48]. In addition, targeting the *MALAT1*/miRNA-200c-3p axis in a xenograft endometrial carcinoma model strongly inhibited tumour growth [49].

Moreover, studies have illustrated that these miRNAs are closely related to melanoma in several ways. miRNA-378a-3p can regulate oncogenic PARVA expression in melanoma, preventing its progression [50]. miRNA-23b-3p was shown to be a tumour suppressor gene in melanoma [51]. miRNA-224-5p can be regulated by E2F1 to drive EMT through TXNIP downregulation in melanoma, and it can inhibit uveal melanoma cell proliferation, migration, and invasion by targeting PIK3R3/AKT3 [52, 53]. miRNA-204-5p, known as a tumour suppressor gene in melanoma, was associated with the CDKN2A pathway and NRAS gene and contributed to BRAF inhibitor resistance [51, 54, 55].^.^ miRNA-205-5p suppresses proliferation and induces senescence via regulation of E2F1 in melanoma [51, 56–58]. miRNA-200b/c-3p act as potential diagnostic and prognostic markers for melanoma [59–61]. Upregulation of miRNA-149-5p, directly regulated by p53, results in increased expression of Mcl-1 and resistance to apoptosis in melanoma cells [62]. Most importantly, studies have confirmed that miRNA-211-5p plays a major role as a tumour suppressor via various targets in melanoma [51, 55, 59, 63, 64]. Moreover, *MALAT1* is an independent risk factor for the prognosis of SKCM according to multivariate Cox regression model analysis. Thus, we believe that *MALAT1* may contribute to the tumorigenesis and survival of SKCM.

Little is known about *LINC00943*. According to the *LINC00943*-miRNA-mRNA subnetwork, miRNA-99a-5p, miRNA-100-5p, miRNA-23b-3p, miRNA-204-5p, miRNA-224-5p, miRNA-149-5p and miRNA-125b-5p closely interacted with *LINC00943*. No connection between *LINC00943* and these miRNAs has been discovered yet; however, these miRNAs were also demonstrated to be associated with melanoma, except miRNA-99a-5p. The links between miRNA-204-5p, miRNA-224-5p, miRNA-149-5p and melanoma are discussed above. In addition, miRNA-23b was suggested as a tumour suppressor gene.^54^ miRNA-100-5p and miRNA-125b-5p are associated with resistance to treatment with immune checkpoint inhibitors in melanoma [65]. Additionally, we confirmed that *LINC00943* is an independent risk factor for the prognosis of SKCM. Therefore, understanding the relationships among *LINC00943*, miRNAs and malignancies may provide further information for future research on melanoma and other malignancies.

Seven KEGG pathways were enriched based on the *LINC00261* subnetwork. One of these pathways, the PI3K/Akt signalling pathway, has been proven to play a critical role in tumorigenesis [66], especially in melanoma [67]. Additionally, a study has demonstrated that *LINC00261* promotes cancer cell proliferation and metastasis in human choriocarcinoma [68]. However, *LINC00261* has shown a strong capacity in improving the chemotherapeutic response and survival of patients with oesophageal cancer [69]. In gastric cancer, *LINC00261* can suppress tumour metastasis by regulating epithelial-mesenchymal transition [70]. Moreover, *LINC00261* can block cellular proliferation by activating the DNA damage response [71]. *LINC00261* may affect the biological behaviour of different tumours in different ways. Therefore, it is essential to further explore the role of *LINC00261* in different tumours. However, five miRNAs, miRNA-23b-3p, miRNA-211-5p, miRNA-205-5p, miRNA-140-3p and miRNA-125b-5p, interacted with *LINC00261* according to the *LINC00261*-miRNA-mRNA subnetwork. Similarly, no connection between *LINC00261* and these miRNAs has been discovered yet. The roles of miRNA-23b-3p, miRNA-211-5p, miRNA-205-5p, and miRNA-125b-5p in melanoma are discussed above. miRNA-140-3p was reported to be regulated by *MALAT1* in uveal melanoma cells [72]. The multivariate Cox regression model for survival suggested that *LINC00261* was not a risk factor for the prognosis of SKCM, however, the median overall survival and disease-free survival time for patients with *LINC00261* CNV deficiency were significantly lower than those without *LINC00261* CNV deficiency (17.03 m vs 61.05 m, 13.50 vs 25.02).

Three of the 16 predicted miRNAs were not associated with *MALAT1, LINC00943* and *LINC00261:* miRNA-21-5p, miRNA-20b-5p and miRNA-424-5p. They are closely related to *SGMS1.AS1, EPB41L4A.AS1* and *SNHG1* according to the ceRNA network. Little is known about miRNA-424-5p in melanoma, while studies have suggested that miRNA-20b-5p may inhibit tumour metastasis via regulation of the PAR-1 receptor in melanoma cells [73], and miRNA-21 may regulate melanoma cell proliferation, migration, and apoptosis through the ERK/NF-κB signalling pathway by targeting SPRY1, PDCD4 and PTEN [74, 75].

## Conclusions

This study advances our understanding of tumorigenesis and development in cutaneous melanoma from the perspective of the ceRNA theory. In addition, *MALAT1* and *LINC00943* may be independent risk factors for the prognosis of patients with cutaneous melanoma and might become predictive molecules for the long-term treatment of melanoma and potential therapeutic targets. Further studies are required to validate the role of *MALAT1*, *LINC00943* and *LINC00261* in cutaneous melanoma.

## Supplementary information

**Additional file 1: Supplementary Table 1.** CNV data and patient information from the Skin Cutaneous Melanoma (TCGA, PanCancer Atlas) [35] and Metastatic Melanoma (DFCI, Science 2015) [36–38].

**Additional file 2: Supplementary Table 2.** Differentially expressed miRNAs in GSE24996、GSE35579、GSE62372.

**Additional file 3: Supplementary Table 3.** Univariate COX regression model for survival analysis of age, sex and stage.

## Data Availability

The data that support results of the present study are available from GEO datasets (including GSE24996(https://www.ncbi.nlm.nih.gov/ geo/query/acc.cgi?acc = GSE24996),GSE35579(https://www.ncbi.nlm.nih.gov/geo/query/acc.cgi?acc=GSE35579),GSE62372(https://www.ncbi.nlm.nih.gov/geo/query/acc.cgi?acc=GSE62372), and GSE112509(https://www.ncbi.nlm.nih.gov/geo/query/ acc.cgi?acc = GSE112509)), and cBioportal (http://www.cbioportal.org/), DAVID (https://david.ncifcrf.gov/), and starbase miRNA-mRNA Interactions (http://starbase.sysu.edu.cn/agoClipRNA.php?source=mRNA), and starbase miRNA-lncRNA Interactions (http://starbase.sysu.edu.cn/agoClipRNA. php?source = lncRNA) database.

## Abbreviations

ceRNA: Competitive endogenous RNA
SKCM: Skin cutaneous melanoma
lncRNA: Long non-coding RNAs
NCBI GEO: National center for biotechnology information gene expression omnibus
GO: Gene ontology
KEGG: Kyoto encyclopedia of genes and genomes
DAVID: Database for annotation, visualization, and integration discovery
MREs: miRNA-response elements
DEMis: Differential expressed miRNAs
DELs: Differential expressed lncRNAs
DEMs: Differential expressed mRNAs
CNV: Copy number variation

## References

[1] McGuire S (2016). World Cancer report 2014. Geneva, Switzerland: World Health Organization, International Agency for Research on Cancer, WHO press, 2015. Adv Nutr.

[2] Berwick M, Erdei E, Hay J (2009). Melanoma epidemiology and public health. Dermatol Clin.

[3] Schadendorf D, van Akkooi ACJ, Berking C, Griewank KG, Gutzmer R, Hauschild A, Stang A, Roesch A, Ugurel S (2018). Melanoma. Lancet.

[4] Disease GBD, Injury I, Prevalence C (2016). Global, regional, and national incidence, prevalence, and years lived with disability for 310 diseases and injuries, 1990-2015: a systematic analysis for the global burden of Disease study 2015. Lancet.

[5] Siegel RL, Miller KD, Jemal A (2020). Cancer statistics, 2020. CA Cancer J Clin.

[6] Burrell RA, McGranahan N, Bartek J, Swanton C (2013). The causes and consequences of genetic heterogeneity in cancer evolution. Nature.

[7] Lander ES, Linton LM, Birren B, Nusbaum C, Zody MC, Baldwin J, Devon K, Dewar K, Doyle M, FitzHugh W (2001). Initial sequencing and analysis of the human genome. Nature.

[8] Yost SE, Smith EN, Schwab RB, Bao L, Jung H, Wang X, Voest E, Pierce JP, Messer K, Parker BA (2012). Identification of high-confidence somatic mutations in whole genome sequence of formalin-fixed breast cancer specimens. Nucleic Acids Res.

[9] Goodrich JA, Kugel JF (2006). Non-coding-RNA regulators of RNA polymerase II transcription. Nat Rev Mol Cell Biol.

[10] Yoon JH, Abdelmohsen K, Gorospe M (2013). Posttranscriptional gene regulation by long noncoding RNA. J Mol Biol.

[11] Kiefer JC (2007). Epigenetics in development. Dev Dyn.

[12] Mikkelsen TS, Ku M, Jaffe DB, Issac B, Lieberman E, Giannoukos G, Alvarez P, Brockman W, Kim TK, Koche RP (2007). Genome-wide maps of chromatin state in pluripotent and lineage-committed cells. Nature.

[13] Joung J, Engreitz JM, Konermann S, Abudayyeh OO, Verdine VK, Aguet F, Gootenberg JS, Sanjana NE, Wright JB, Fulco CP (2017). Genome-scale activation screen identifies a lncRNA locus regulating a gene neighbourhood. Nature.

[14] Leucci E, Vendramin R, Spinazzi M, Laurette P, Fiers M, Wouters J, Radaelli E, Eyckerman S, Leonelli C, Vanderheyden K (2016). Melanoma addiction to the long non-coding RNA SAMMSON. Nature.

[15] Hosono Y, Niknafs YS, Prensner JR, Iyer MK, Dhanasekaran SM, Mehra R, Pitchiaya S, Tien J, Escara-Wilke J, Poliakov A (2017). Oncogenic role of THOR, a conserved Cancer/testis long non-coding RNA. Cell.

[16] Montes M, Nielsen MM, Maglieri G, Jacobsen A, Hojfeldt J, Agrawal-Singh S, Hansen K, Helin K, van de Werken HJG, Pedersen JS (2015). The lncRNA MIR31HG regulates p16(INK4A) expression to modulate senescence. Nat Commun.

[17] Li P, He J, Yang Z, Ge S, Zhang H, Zhong Q, Fan X (2020). ZNNT1 long noncoding RNA induces autophagy to inhibit tumorigenesis of uveal melanoma by regulating key autophagy gene expression. Autophagy.

[18] Jalali S, Bhartiya D, Lalwani MK, Sivasubbu S, Scaria V (2013). Systematic transcriptome wide analysis of lncRNA-miRNA interactions. PLoS One.

[19] Ala U, Karreth FA, Bosia C, Pagnani A, Taulli R, Leopold V, Tay Y, Provero P, Zecchina R, Pandolfi PP (2013). Integrated transcriptional and competitive endogenous RNA networks are cross-regulated in permissive molecular environments. Proc Natl Acad Sci U S A.

[20] Salmena L, Poliseno L, Tay Y, Kats L, Pandolfi PP (2011). A ceRNA hypothesis: the Rosetta stone of a hidden RNA language?. Cell.

[21] Tay Y, Rinn J, Pandolfi PP (2014). The multilayered complexity of ceRNA crosstalk and competition. Nature.

[22] Rinn JL, Chang HY (2012). Genome regulation by long noncoding RNAs. Annu Rev Biochem.

[23] Liu XH, Sun M, Nie FQ, Ge YB, Zhang EB, Yin DD, Kong R, Xia R, Lu KH, Li JH (2014). Lnc RNA HOTAIR functions as a competing endogenous RNA to regulate HER2 expression by sponging miR-331-3p in gastric cancer. Mol Cancer.

[24] Zhong Z, Huang M, Lv M, He Y, Duan C, Zhang L, Chen J (2017). Circular RNA MYLK as a competing endogenous RNA promotes bladder cancer progression through modulating VEGFA/VEGFR2 signaling pathway. Cancer Lett.

[25] Chang L, Guo R, Yuan Z, Shi H, Zhang D (2018). LncRNA HOTAIR regulates CCND1 and CCND2 expression by sponging miR-206 in ovarian Cancer. Cell Physiol Biochem.

[26] Edgar R, Domrachev M, Lash AE (2002). Gene expression omnibus: NCBI gene expression and hybridization array data repository. Nucleic Acids Res.

[27] Breuer J: R (Software). In., edn.; 2017.

[28] Smyth GK: limma: Linear Models for Microarray Data. 2005.

[29] Love MI, Huber W, Anders S (2014). Moderated estimation of fold change and dispersion for RNA-seq data with DESeq2. Genome Biol.

[30] Li JH, Liu S, Zhou H, Qu LH, Yang JH (2014). starBase v2.0: decoding miRNA-ceRNA, miRNA-ncRNA and protein-RNA interaction networks from large-scale CLIP-Seq data. Nucleic Acids Res.

[31] Liao Q, Liu C, Yuan X, Kang S, Miao R, Xiao H, Zhao G, Luo H, Bu D, Zhao H (2011). Large-scale prediction of long non-coding RNA functions in a coding-non-coding gene co-expression network. Nucleic Acids Res.

[32] Shannon P, Markiel A, Ozier O, Baliga NS, Wang JT, Ramage D, Amin N, Schwikowski B, Ideker T (2003). Cytoscape: a software environment for integrated models of biomolecular interaction networks. Genome Res.

[33] Huang da W, Sherman BT, Lempicki RA (2009). Bioinformatics enrichment tools: paths toward the comprehensive functional analysis of large gene lists. Nucleic Acids Res.

[34] Huang da W, Sherman BT, Lempicki RA (2009). Systematic and integrative analysis of large gene lists using DAVID bioinformatics resources. Nat Protoc.

[35] Hoadley KA, Yau C, Hinoue T, Wolf DM, Lazar AJ, Drill E, Shen R, Taylor AM, Cherniack AD, Thorsson V (2018). Cell-of-origin patterns dominate the molecular classification of 10,000 tumors from 33 types of Cancer. Cell.

[36] Van Allen EM, Miao D, Schilling B, Shukla SA, Blank C, Zimmer L, Sucker A, Hillen U, Foppen MHG, Goldinger SM (2015). Genomic correlates of response to CTLA-4 blockade in metastatic melanoma. Science.

[37] Tryka KA, Hao L, Sturcke A, Jin Y, Wang ZY, Ziyabari L, Lee M, Popova N, Sharopova N, Kimura M (2014). NCBI's database of genotypes and phenotypes: dbGaP. Nucleic Acids Res.

[38] Mailman MD, Feolo M, Jin Y, Kimura M, Tryka K, Bagoutdinov R, Hao L, Kiang A, Paschall J, Phan L (2007). The NCBI dbGaP database of genotypes and phenotypes. Nat Genet.

[39] Gao J, Aksoy BA, Dogrusoz U, Dresdner G, Gross B, Sumer SO, Sun Y, Jacobsen A, Sinha R, Larsson E (2013). Integrative analysis of complex cancer genomics and clinical profiles using the cBioPortal. Sci Signal.

[40] Gutschner T, Hammerle M, Eissmann M, Hsu J, Kim Y, Hung G, Revenko A, Arun G, Stentrup M, Gross M (2013). The noncoding RNA MALAT1 is a critical regulator of the metastasis phenotype of lung cancer cells. Cancer Res.

[41] Gutschner T, Hammerle M, Diederichs S (2013). MALAT1 -- a paradigm for long noncoding RNA function in cancer. J Mol Med (Berl).

[42] Lai MC, Yang Z, Zhou L, Zhu QQ, Xie HY, Zhang F, Wu LM, Chen LM, Zheng SS (2012). Long non-coding RNA MALAT-1 overexpression predicts tumor recurrence of hepatocellular carcinoma after liver transplantation. Med Oncol.

[43] Fujimoto A, Furuta M, Totoki Y, Tsunoda T, Kato M, Shiraishi Y, Tanaka H, Taniguchi H, Kawakami Y, Ueno M (2016). Whole-genome mutational landscape and characterization of noncoding and structural mutations in liver cancer. Nat Genet.

[44] YiRen H, YingCong Y, Sunwu Y, Keqin L, Xiaochun T, Senrui C, Ende C, XiZhou L, Yanfan C (2017). Long noncoding RNA MALAT1 regulates autophagy associated chemoresistance via miR-23b-3p sequestration in gastric cancer. Mol Cancer.

[45] Tao F, Tian X, Ruan S, Shen M, Zhang Z. miR-211 sponges lncRNA MALAT1 to suppress tumor growth and progression through inhibiting PHF19 in ovarian carcinoma. FASEB J. 2018;32:fj.201800495RR. 10.1096/fj.201800495RR 29874124

[46] Hirata H, Hinoda Y, Shahryari V, Deng G, Nakajima K, Tabatabai ZL, Ishii N, Dahiya R (2015). Long noncoding RNA MALAT1 promotes aggressive renal cell carcinoma through Ezh2 and interacts with miR-205. Cancer Res.

[47] Li J, Wang J, Chen Y, Li S, Jin M, Wang H, Chen Z, Yu W (2016). LncRNA MALAT1 exerts oncogenic functions in lung adenocarcinoma by targeting miR-204. Am J Cancer Res.

[48] Tan X, Huang Z, Li X (2017). Long non-coding RNA MALAT1 interacts with miR-204 to modulate human Hilar Cholangiocarcinoma proliferation, migration, and invasion by targeting CXCR4. J Cell Biochem.

[49] Li Q, Zhang C, Chen R, Xiong H, Qiu F, Liu S, Zhang M, Wang F, Wang Y, Zhou X (2016). Disrupting MALAT1/miR-200c sponge decreases invasion and migration in endometrioid endometrial carcinoma. Cancer Lett.

[50] Velazquez-Torres G, Shoshan E, Ivan C, Huang L, Fuentes-Mattei E, Paret H, Kim SJ, Rodriguez-Aguayo C, Xie V, Brooks D (2018). A-to-I miR-378a-3p editing can prevent melanoma progression via regulation of PARVA expression. Nat Commun.

[51] Kozubek J, Ma Z, Fleming E, Duggan T, Wu R, Shin DG, Dadras SS (2013). In-depth characterization of microRNA transcriptome in melanoma. PLoS One.

[52] Knoll S, Furst K, Kowtharapu B, Schmitz U, Marquardt S, Wolkenhauer O, Martin H, Putzer BM (2014). E2F1 induces miR-224/452 expression to drive EMT through TXNIP downregulation. EMBO Rep.

[53] Li J, Liu X, Li C, Wang W (2019). miR-224-5p inhibits proliferation, migration, and invasion by targeting PIK3R3/AKT3 in uveal melanoma. J Cell Biochem.

[54] Galasso M, Morrison C, Minotti L, Corra F, Zerbinati C, Agnoletto C, Baldassari F, Fassan M, Bartolazzi A, Vecchione A (2018). Loss of miR-204 expression is a key event in melanoma. Mol Cancer.

[55] Diaz-Martinez M, Benito-Jardon L, Alonso L, Koetz-Ploch L, Hernando E, Teixido J (2018). miR-204-5p and miR-211-5p contribute to BRAF inhibitor resistance in melanoma. Cancer Res.

[56] Xu Y, Brenn T, Brown ER, Doherty V, Melton DW (2012). Differential expression of microRNAs during melanoma progression: miR-200c, miR-205 and miR-211 are downregulated in melanoma and act as tumour suppressors. Br J Cancer.

[57] Dar AA, Majid S, de Semir D, Nosrati M, Bezrookove V (2011). Kashani-Sabet M: **miRNA-205 suppresses melanoma cell proliferation and induces senescence via regulation of E2F1 protein**. J Biol Chem.

[58] Sanchez-Sendra B, Martinez-Ciarpaglini C, Gonzalez-Munoz JF, Murgui A, Terradez L, Monteagudo C (2018). Downregulation of intratumoral expression of miR-205, miR-200c and miR-125b in primary human cutaneous melanomas predicts shorter survival. Sci Rep.

[59] Mirzaei H, Gholamin S, Shahidsales S, Sahebkar A, Jaafari MR, Mirzaei HR, Hassanian SM, Avan A (2016). MicroRNAs as potential diagnostic and prognostic biomarkers in melanoma. Eur J Cancer.

[60] Elson-Schwab I, Lorentzen A, Marshall CJ. MicroRNA-200 family members differentially regulate morphological plasticity and mode of melanoma cell invasion. PLoS One. 2010;5(10):e13176. 10.1371/journal.pone.0013176 PMC2949394 20957176

[61] Zhao H, Xing G, Wang Y, Luo Z, Liu G, Meng H. Long noncoding RNA HEIH promotes melanoma cell proliferation, migration and invasion via inhibiting miR-200b/a/429. Biosci Rep. 2017;37:BSR20170682. 10.1042/BSR20170682 PMC5479024 28487474

[62] Jin L, Hu WL, Jiang CC, Wang JX, Han CC, Chu P, Zhang LJ, Thorne RF, Wilmott J, Scolyer RA (2011). MicroRNA-149*, a p53-responsive microRNA, functions as an oncogenic regulator in human melanoma. Proc Natl Acad Sci U S A.

[63] Bell RE, Khaled M, Netanely D, Schubert S, Golan T, Buxbaum A, Janas MM, Postolsky B, Goldberg MS, Shamir R (2014). Transcription factor/microRNA axis blocks melanoma invasion program by miR-211 targeting NUAK1. J Invest Dermatol.

[64] Levy C, Khaled M, Iliopoulos D, Janas MM, Schubert S, Pinner S, Chen PH, Li S, Fletcher AL, Yokoyama S (2010). Intronic miR-211 assumes the tumor suppressive function of its host gene in melanoma. Mol Cell.

[65] Huber V, Vallacchi V, Fleming V, Hu X, Cova A, Dugo M, Shahaj E, Sulsenti R, Vergani E, Filipazzi P (2018). Tumor-derived microRNAs induce myeloid suppressor cells and predict immunotherapy resistance in melanoma. J Clin Invest.

[66] Fruman DA, Rommel C (2014). PI3K and cancer: lessons, challenges and opportunities. Nat Rev Drug Discov.

[67] Davies MA (2012). The role of the PI3K-AKT pathway in melanoma. Cancer J.

[68] Wang Y, Xue K, Guan Y, Jin Y, Liu S, Wang Y, Liu S, Wang L, Han L (2017). Long noncoding RNA LINC00261 suppresses cell proliferation and invasion and promotes cell apoptosis in human Choriocarcinoma. Oncol Res.

[69] Lin K, Jiang H, Zhuang SS, Qin YS, Qiu GD, She YQ, Zheng JT, Chen C, Fang L, Zhang SY (2019). Long noncoding RNA LINC00261 induces chemosensitization to 5-fluorouracil by mediating methylation-dependent repression of DPYD in human esophageal cancer. FASEB J.

[70] Yu Y, Li L, Zheng Z, Chen S, Chen E, Hu Y (2017). Long non-coding RNA linc00261 suppresses gastric cancer progression via promoting slug degradation. J Cell Mol Med.

[71] Shahabi S, Kumaran V, Castillo J, Cong Z, Nandagopal G, Mullen DJ, Alvarado A, Correa MR, Saizan A, Goel R (2019). LINC00261 is an epigenetically regulated tumor suppressor essential for activation of the DNA damage response. Cancer Res.

[72] Sun L, Sun P, Zhou QY, Gao X, Han Q (2016). Long noncoding RNA MALAT1 promotes uveal melanoma cell growth and invasion by silencing of miR-140. Am J Transl Res.

[73] Saleiban A, Faxalv L, Claesson K, Jonsson JI, Osman A (2014). miR-20b regulates expression of proteinase-activated receptor-1 (PAR-1) thrombin receptor in melanoma cells. Pigment Cell Melanoma Res.

[74] Mao XH, Chen M, Wang Y, Cui PG, Liu SB, Xu ZY (2017). MicroRNA-21 regulates the ERK/NF-kappaB signaling pathway to affect the proliferation, migration, and apoptosis of human melanoma A375 cells by targeting SPRY1, PDCD4, and PTEN. Mol Carcinog.

[75] Yang CH, Yue J, Pfeffer SR, Handorf CR, Pfeffer LM (2011). MicroRNA miR-21 regulates the metastatic behavior of B16 melanoma cells. J Biol Chem.

