# Supplementary material for: Reconstruction of lncRNA-miRNA-mRNA network based on competitive endogenous RNA reveals functional lncRNAs in skin cutaneous melanoma
Source: BMC Cancer. 2020 Sep 29;20:927. doi: 10.1186/s12885-020-07302-5 (PMC7523354; doi:10.1186/s12885-020-07302-5)
Supplement: Supplementary file 1 — Additional file 1: Supplementary Table 1. CNV data and patient information from the Skin Cutaneous Melanoma (TCGA, PanCancer Atlas) [35] and Metastatic Melanoma (DFCI, Science 2015) [36–38]. [file 12885_2020_7302_MOESM1_ESM.docx]

| Patient ID^a^ | *MALAT1* CNV status^b^ | *LINC00943* CNV status^c^ | *LINC00261* CNV status^d^ | Age^e^ | Gender^f^ | Stage^g^ | OS Status^h^ | OS (months) | DFS Status^i^ | DFS (months) |
| --- | --- | --- | --- | --- | --- | --- | --- | --- | --- | --- |
| TCGA-3N-A9WB | 0 | 0 | 1 |  | 0 | 1 | 1 | 17.03 | 1 | 14.01 |
| TCGA-3N-A9WC | 1 | 0 | 0 |  | 0 | 2 | 0 | 66.48 | 1 | 56.05 |
| TCGA-3N-A9WD | 0 | 0 | 0 |  | 0 | 3 | 1 | 12.99 | 1 | 10.06 |
| TCGA-BF-A1PU | 0 | 1 | 0 |  | 1 | 2 | 0 | 12.72 | 1 | 15.91 |
| TCGA-BF-A1PV | 0 | 0 | 0 |  | 1 | 2 | 0 | 0.46 | 0 | 0.46 |
| TCGA-BF-A1PX | 0 | 0 | 0 |  | 0 | 3 | 1 | 9.27 | 0 | 9.27 |
| TCGA-BF-A1PZ | 0 | 0 | 0 |  | 1 | 2 | 0 | 28.04 | 0 | 28.04 |
| TCGA-BF-A1Q0 | 0 | 0 | 0 |  | 0 | 2 | 0 | 27.32 | 0 | 27.32 |
| TCGA-BF-A3DJ | 0 | 0 | 0 |  | 1 | 3 | 0 | 15.25 | 1 | 7.13 |
| TCGA-BF-A3DL | 0 | 0 | 0 |  | 1 | 3 | 0 | 25.28 | 0 | 25.28 |
| TCGA-BF-A3DM | 0 | 0 | 0 |  | 0 | 2 | 0 | 19.76 | 0 | 19.76 |
| TCGA-BF-A3DN | 0 | 0 | 0 |  | 1 | 3 | 0 | 23.57 | 0 | 23.57 |
| TCGA-BF-A5EO | 0 | 0 | 0 |  | 0 | 2 | 0 | 23.11 | 0 | 23.11 |
| TCGA-BF-A5EP | 0 | 0 | 0 |  | 1 | 3 | 0 | 11.01 | 0 | 11.01 |
| TCGA-BF-A5EQ | 0 | 0 | 0 |  | 0 | 2 | 0 | 10.62 | 0 | 10.62 |
| TCGA-BF-A5ER | 0 | 0 | 0 |  | 0 | 2 | 0 | 10.75 | 0 | 10.75 |
| TCGA-BF-A5ES | 0 | 0 | 0 |  | 1 | 2 | 0 | 16.11 | 0 | 16.11 |
| TCGA-BF-AAP0 | 0 | 0 | 0 |  | 1 | 4 | 0 | 14.93 | 0 | 14.93 |
| TCGA-D3-A1Q1 | 1 | 0 | 0 |  | 1 | 3 | 1 | 16.57 | 1 | 1.91 |
| TCGA-D3-A1Q3 | 1 | 0 | 0 |  | 0 | 2 | 1 | 16.67 | 1 | 16.67 |
| TCGA-D3-A1Q4 | 0 | 0 | 0 |  | 1 | 3 | 0 | 112.04 | 0 | 112.04 |
| TCGA-D3-A1Q5 | 1 | 0 | 0 |  | 0 | 2 | 1 | 112.57 | 1 | 80.61 |
| TCGA-D3-A1Q6 | 0 | 0 | 0 |  | 0 | 3 | 1 | 71.8 | 1 | 1.58 |
| TCGA-D3-A1Q7 | 0 | 0 | 0 |  | 1 | 1 | 0 | 133.25 | 1 | 30.28 |
| TCGA-D3-A1Q8 | 0 | 0 | 0 |  | 0 | 4 | 1 | 28.08 | 1 | 4.83 |
| TCGA-D3-A1Q9 | 0 | 0 | 0 |  | 0 | 3 | 1 | 31.59 | 1 | 1.45 |
| TCGA-D3-A1QA | 0 | 1 | 0 |  | 0 | 1 | 0 | 90.9 | 1 | 45.8 |
| TCGA-D3-A1QB | 0 | 0 | 0 |  | 1 | 3 | 0 | 95.74 | *N/A* | *N/A* |
| TCGA-D3-A2J6 | 1 | 1 | 0 |  | 0 | 2 | 1 | 43.43 | 1 | 14.73 |
| TCGA-D3-A2J7 | 0 | 0 | 0 |  | 0 | 3 | 1 | 103.1 | 1 | 0.43 |
| TCGA-D3-A2J8 | 0 | 0 | 0 |  | 0 | 1 | 1 | 65.49 | 1 | 57.11 |
| TCGA-D3-A2J9 | 0 | 0 | 0 |  | 0 | 3 | 1 | 23.77 | 1 | 7.59 |
| TCGA-D3-A2JA | 0 | 0 | 0 |  | 0 | 3 | 0 | 115.53 | 1 | 1.32 |
| TCGA-D3-A2JC | 0 | 0 | 0 |  | 1 | 3 | 0 | 86.76 | 0 | 86.76 |
| TCGA-D3-A2JD | 0 | 0 | 0 |  | 0 | 3 | 1 | 11.87 | 1 | 2.79 |
| TCGA-D3-A2JE | 0 | 0 | 0 |  | 1 | 3 | 1 | 27.65 | 1 | 4.9 |
| TCGA-D3-A2JF | 0 | 0 | 0 |  | 0 | 1 | 0 | 62.07 | 1 | 0.82 |
| TCGA-D3-A2JG | 0 | 0 | 0 |  | 1 | 3 | 1 | 113.52 | 1 | 107.8 |
| TCGA-D3-A2JH | 0 | 0 | 0 |  | 0 | 1 | 0 | 42.08 | 1 | 18.71 |
| TCGA-D3-A2JK | 0 | 0 | 0 |  | 0 | 3 | 1 | 12.1 | 1 | 8.58 |
| TCGA-D3-A2JL | 0 | 0 | 0 |  | 1 | 2 | 0 | 171.58 | 1 | 86.73 |
| TCGA-D3-A2JN | 0 | 0 | 0 |  | 1 | 3 | 1 | 66.48 | 0 | 66.48 |
| TCGA-D3-A2JO | 0 | 0 | 0 |  | 1 | 3 | 0 | 66.08 | 1 | 1.64 |
| TCGA-D3-A2JP | 0 | 0 | 0 |  | 0 | 3 | 0 | 59.57 | 1 | 7.46 |
| TCGA-D3-A3BZ | 0 | 0 | 0 |  | 0 | 2 | 0 | 130.72 | 1 | 76.93 |
| TCGA-D3-A3C6 | 0 | 1 | 0 |  | 1 | 1 | 1 | 58.06 | 1 | 47.37 |
| TCGA-D3-A3C7 | 1 | 0 | 0 |  | 1 | 3 | 0 | 46.98 | 0 | 46.98 |
| TCGA-D3-A3C8 | 0 | 0 | 0 |  | 1 | 3 | 0 | 46.32 | 1 | 1.81 |
| TCGA-D3-A3CB | 0 | 0 | 0 |  | 0 | 2 | 0 | 166.52 | 0 | 166.52 |
| TCGA-D3-A3CC | 0 | 1 | 0 |  | 1 | 2 | 0 | 86.93 | 1 | 3.16 |
| TCGA-D3-A3CE | 0 | 0 | 0 |  | 1 | 3 | 1 | 60.23 | *N/A* | *N/A* |
| TCGA-D3-A3CF | 0 | 0 | 0 |  | 1 | 3 | 1 | 24.53 | 1 | 4.24 |
| TCGA-D3-A3ML | 0 | 0 | 0 |  | 0 | 3 | 1 | 13.87 | 1 | 1.05 |
| TCGA-D3-A3MO | 0 | 0 | 0 |  | 0 | 3 | 1 | 9.34 | 1 | 1.55 |
| TCGA-D3-A3MR | 0 | 0 | 0 |  | 0 | 3 | 0 | 103.59 | 1 | 102.18 |
| TCGA-D3-A3MU | 0 | 0 | 0 |  | 0 | 3 | 0 | 39.75 | 1 | 24.66 |
| TCGA-D3-A3MV | 0 | 0 | 0 |  | 1 | 3 | 0 | 45.3 | 1 | 0.53 |
| TCGA-D3-A51E | 1 | 0 | 0 |  | 1 | 2 | 0 | 174.84 | 0 | 174.84 |
| TCGA-D3-A51F | 0 | 0 | 0 |  | 0 | 3 | 0 | 55.73 | 0 | 55.73 |
| TCGA-D3-A51H | 0 | 0 | 0 |  | 0 | 3 | 0 | 56.35 | 1 | 27.55 |
| TCGA-D3-A51J | 0 | 0 | 0 |  | 0 | 3 | 0 | 145.12 | 1 | 66.02 |
| TCGA-D3-A51K | 0 | 0 | 0 |  | 0 | 3 | 0 | 32.94 | 0 | 32.94 |
| TCGA-D3-A51N | 0 | 0 | 0 |  | 1 | 4 | 0 | 22.62 | 1 | 0.59 |
| TCGA-D3-A51R | 0 | 0 | 0 |  | 0 | 2 | 0 | 63.81 | 1 | 33.4 |
| TCGA-D3-A51T | 0 | 0 | 0 |  | 1 | 3 | 0 | 26.89 | 1 | 6.9 |
| TCGA-D3-A5GL | 0 | 0 | 0 |  | 0 | 1 | 0 | 125.78 | 1 | 35.9 |
| TCGA-D3-A5GN | 1 | 0 | 0 |  | 1 | 1 | 0 | 135.75 | 1 | 42.05 |
| TCGA-D3-A5GO | 0 | 0 | 0 |  | 0 | 2 | 0 | 137.92 | 1 | 24.89 |
| TCGA-D3-A5GR | 0 | 0 | 0 |  | 1 | 3 | 0 | 178.32 | *N/A* | *N/A* |
| TCGA-D3-A5GS | 1 | 0 | 0 |  | 0 | 4 | 0 | 18.18 | 1 | 1.45 |
| TCGA-D3-A5GT | 0 | 0 | 0 |  | 0 | 3 | 0 | 16.01 | 1 | 9.57 |
| TCGA-D3-A5GU | 1 | 0 | 0 |  | 0 | 1 | 0 | 125.19 | 1 | 101.06 |
| TCGA-D3-A8GB | 0 | 0 | 0 |  | 0 | 3 | 1 | 30.84 | 1 | 11.41 |
| TCGA-D3-A8GC | 1 | 1 | 0 |  | 0 | 3 | 1 | 79.59 | 1 | 61.12 |
| TCGA-D3-A8GD | 0 | 0 | 0 |  | 1 | 3 | 0 | 23.61 | 1 | 4.83 |
| TCGA-D3-A8GE | 0 | 0 | 0 |  | 0 | 4 | 0 | 26.43 | 1 | 1.94 |
| TCGA-D3-A8GI | 0 | 0 | 0 |  | 0 | 1 | 1 | 58.52 | 1 | 1.64 |
| TCGA-D3-A8GJ | 0 | 0 | 0 |  | 0 | 2 | 0 | 241.38 | 1 | 47.44 |
| TCGA-D3-A8GK | 0 | 0 | 1 |  | 0 | 2 | 0 | 170.2 | 1 | 51.12 |
| TCGA-D3-A8GL | 0 | 1 | 0 |  | 0 | 3 | 1 | 89.13 | 1 | 43.82 |
| TCGA-D3-A8GM | 0 | 0 | 0 |  | 0 | 2 | 1 | 107.14 | 0 | 107.14 |
| TCGA-D3-A8GN | 0 | 0 | 0 |  | 1 | 2 | 0 | 161 | 1 | 25.87 |
| TCGA-D3-A8GP | 1 | 0 | 0 |  | 0 | 3 | 0 | 152.74 | 1 | 17.59 |
| TCGA-D3-A8GQ | 0 | 0 | 0 |  | 0 | 2 | 1 | 29.06 | 1 | 19 |
| TCGA-D3-A8GS | 0 | 0 | 0 |  | 0 | 1 | 1 | 117.17 | 1 | 86.83 |
| TCGA-D3-A8GV | 0 | 0 | 0 |  | 0 | 2 | 1 | 167.7 | 1 | 128.09 |
| TCGA-D9-A148 | 0 | 0 | 0 |  | 0 | *N/A* | 0 | 151.53 | 1 | 140.94 |
| TCGA-D9-A149 | 0 | 0 | 0 |  | 1 | *N/A* | 0 | 54.67 | 1 | 42.41 |
| TCGA-D9-A1JW | 0 | 0 | 0 |  | 0 | *N/A* | 0 | 3.65 | 1 | 0.33 |
| TCGA-D9-A1JX | 1 | 0 | 0 |  | 1 | *N/A* | 1 | 7.1 | 1 | 6.41 |
| TCGA-D9-A1X3 | 0 | 0 | 0 |  | 0 | *N/A* | 0 | 18.11 | 0 | 18.11 |
| TCGA-D9-A3Z1 | 0 | 0 | 0 |  | 0 | 3 | 1 | 15.39 | 1 | 11.01 |
| TCGA-D9-A3Z3 | 1 | 0 | 0 |  | 1 | 3 | 0 | 22.29 | 0 | 22.29 |
| TCGA-D9-A3Z4 | 1 | 0 | 0 |  | 0 | 3 | 1 | 17.06 | 1 | 6.31 |
| TCGA-D9-A4Z2 | 0 | 0 | 0 |  | 0 | 3 | 1 | 6.25 | 1 | 2.63 |
| TCGA-D9-A4Z3 | 0 | 0 | 0 |  | 1 | 3 | 0 | 16.6 | 1 | 3.29 |
| TCGA-D9-A4Z5 | 1 | 1 | 0 |  | 0 | 2 | 0 | 7.17 | 0 | 7.17 |
| TCGA-D9-A4Z6 | 0 | 0 | 0 |  | 0 | 3 | 1 | 18.44 | 1 | 7 |
| TCGA-D9-A6E9 | 0 | 0 | 0 |  | 1 | 3 | 0 | 9.9 | 0 | 9.9 |
| TCGA-D9-A6EA | 0 | 1 | 0 |  | 0 | 3 | 0 | 25.18 | 0 | 25.18 |
| TCGA-D9-A6EC | 0 | 1 | 0 |  | 0 | 3 | 0 | 77.56 | 0 | 77.56 |
| TCGA-D9-A6EG | 1 | 0 | 0 |  | 0 | 3 | 1 | 22.95 | 1 | 13.08 |
| TCGA-DA-A1HV | 0 | 0 | 0 |  | 1 | 3 | 0 | 76.57 | 1 | 28.47 |
| TCGA-DA-A1HW | 0 | 0 | 0 |  | 1 | 3 | 0 | 26.96 | 1 | 28.01 |
| TCGA-DA-A1HY | 0 | 0 | 0 |  | 0 | 3 | 0 | 144.89 | 0 | 144.89 |
| TCGA-DA-A1I0 | 0 | 0 | 0 |  | 0 | 4 | 1 | 20.38 | 1 | 19.69 |
| TCGA-DA-A1I1 | 0 | 0 | 0 |  | 0 | 3 | 0 | 222.51 | 1 | 183.81 |
| TCGA-DA-A1I2 | 0 | 0 | 0 |  | 0 | 3 | 1 | 176.55 | 1 | 153.83 |
| TCGA-DA-A1I4 | 0 | 0 | 0 |  | 0 | 3 | 1 | 35.93 | 1 | 29.23 |
| TCGA-DA-A1I5 | 0 | 0 | 0 |  | 1 | 4 | 0 | 135.02 | 0 | 135.02 |
| TCGA-DA-A1I7 | 0 | 0 | 0 |  | 0 | 3 | 0 | 88.86 | 1 | 24.82 |
| TCGA-DA-A1I8 | 0 | 0 | 0 |  | 1 | 2 | 1 | 53.92 | 1 | 31.59 |
| TCGA-DA-A1IA | 0 | 1 | 0 |  | 1 | 3 | 1 | 65.92 | 1 | 65.92 |
| TCGA-DA-A1IB | 0 | 0 | 0 |  | 1 | 3 | 0 | 27.12 | 1 | 7.99 |
| TCGA-DA-A1IC | 0 | 0 | 0 |  | 0 | 3 | 1 | 68.09 | 1 | 63.45 |
| TCGA-DA-A3F2 | 0 | 0 | 0 |  | 0 | 3 | 1 | 33.93 | 1 | 14.14 |
| TCGA-DA-A3F3 | 0 | 0 | 0 |  | 0 | 3 | 1 | 10.49 | 1 | 10.49 |
| TCGA-DA-A3F5 | 0 | 0 | 0 |  | 0 | 1 | 1 | 225.96 | 1 | 224.91 |
| TCGA-DA-A3F8 | 0 | 0 | 0 |  | 0 | 3 | 0 | 43.36 | 0 | 43.36 |
| TCGA-DA-A95V | 0 | 0 | 0 |  | 1 | 2 | 0 | 72.1 | 0 | 72.1 |
| TCGA-DA-A95W | 0 | 0 | 0 |  | 0 | 3 | 0 | 37.35 | 0 | 37.35 |
| TCGA-DA-A95X | 0 | 1 | 0 |  | 0 | 1 | 0 | 73.94 | 0 | 73.94 |
| TCGA-DA-A95Y | 0 | 1 | 1 |  | 0 | 2 | 1 | 14.14 | 1 | 11.11 |
| TCGA-DA-A95Z | 0 | 0 | 0 |  | 0 | 4 | 0 | 13.02 | 0 | 13.02 |
| TCGA-EB-A1NK | 0 | 1 | 0 |  | 0 | 2 | 0 | 34.16 | 0 | 34.16 |
| TCGA-EB-A24C | 0 | 1 | 0 |  | 0 | *N/A* | 0 | 20.78 | 1 | 15.29 |
| TCGA-EB-A24D | 1 | 0 | 0 |  | 0 | 3 | 0 | 21.21 | 1 | 16.44 |
| TCGA-EB-A299 | 0 | 0 | 0 |  | 0 | 2 | 0 | 12.43 | 0 | 12.43 |
| TCGA-EB-A3HV | 0 | 1 | 0 |  | 0 | 2 | 0 | 1.28 | 0 | 1.28 |
| TCGA-EB-A3XB | 0 | 0 | 0 |  | 0 | 2 | 0 | 26.17 | 0 | 26.17 |
| TCGA-EB-A3XC | 0 | 0 | 0 |  | 0 | 2 | 0 | 21.37 | 1 | 2.43 |
| TCGA-EB-A3XD | 0 | 0 | 0 |  | 1 | 2 | 0 | 38.14 | 0 | 38.14 |
| TCGA-EB-A3XE | 0 | 1 | 0 |  | 1 | 2 | 0 | 5.92 | 0 | 5.92 |
| TCGA-EB-A3XF | 0 | 0 | 0 |  | 0 | 2 | 0 | 9.14 | 0 | 9.14 |
| TCGA-EB-A3Y6 | 0 | 0 | 0 |  | 1 | 2 | 0 | 4.14 | 0 | 4.14 |
| TCGA-EB-A3Y7 | 1 | 0 | 0 |  | 1 | 3 | 1 | 10.72 | 0 | 10.72 |
| TCGA-EB-A41A | 0 | 0 | 0 |  | 0 | 2 | 0 | 8.61 | 0 | 8.61 |
| TCGA-EB-A41B | 1 | 0 | 0 |  | 1 | 2 | 0 | 9.57 | 0 | 9.57 |
| TCGA-EB-A42Y | 0 | 0 | 0 |  | 1 | 2 | 1 | 23.7 | 1 | 23.7 |
| TCGA-EB-A42Z | 0 | 1 | 0 |  | 0 | 3 | 0 | 14.5 | 0 | 14.5 |
| TCGA-EB-A431 | 0 | 0 | 0 |  | 0 | 2 | 0 | 18.67 | 0 | 18.67 |
| TCGA-EB-A44N | 0 | 0 | 0 |  | 0 | 2 | 1 | 6.74 | 1 | 3.78 |
| TCGA-EB-A44O | 1 | 0 | 0 |  | 0 | 2 | 0 | 2.66 | 1 | 1.97 |
| TCGA-EB-A44P | 0 | 0 | 0 |  | 1 | 2 | 0 | 24.36 | 0 | 24.36 |
| TCGA-EB-A44Q | 0 | 0 | 0 |  | 1 | 3 | 0 | 13.87 | 0 | 13.87 |
| TCGA-EB-A44R | 1 | 0 | 0 |  | 0 | 3 | 1 | 10.36 | 1 | 10.36 |
| TCGA-EB-A4IQ | 0 | 0 | 0 |  | 1 | 3 | 1 | 20.91 | 1 | 11.08 |
| TCGA-EB-A4IS | 0 | 0 | 0 |  | 0 | 2 | 0 | 25.45 | 0 | 25.45 |
| TCGA-EB-A4OY | 0 | 0 | 0 |  | 1 | 3 | 0 | 32.12 | 0 | 32.12 |
| TCGA-EB-A4OZ | 0 | 0 | 0 |  | 1 | 3 | 0 | 20.38 | 0 | 20.38 |
| TCGA-EB-A4P0 | 0 | 0 | 0 |  | 0 | 2 | 1 | 10.72 | 0 | 10.72 |
| TCGA-EB-A4XL | 0 | 0 | 0 |  | 1 | 2 | 0 | 25.54 | 0 | 25.54 |
| TCGA-EB-A551 | 0 | 0 | 0 |  | 1 | 3 | 0 | 19.4 | 0 | 19.4 |
| TCGA-EB-A553 | 0 | 0 | 0 |  | 0 | 2 | 0 | 7.43 | 0 | 7.43 |
| TCGA-EB-A57M | 1 | 0 | 0 |  | 0 | 3 | 1 | 15.52 | 1 | 15.52 |
| TCGA-EB-A5FP | 0 | 0 | 0 |  | 1 | 4 | 1 | 14.93 | 1 | 0.79 |
| TCGA-EB-A5KH | 0 | 0 | 0 |  | 0 | 3 | 1 | 20.35 | 0 | 20.35 |
| TCGA-EB-A5SE | 0 | 0 | 0 |  | 0 | 2 | 1 | 13.18 | 1 | 12.07 |
| TCGA-EB-A5SF | 0 | 0 | 0 |  | 1 | 2 | 1 | 12.13 | 0 | 12.13 |
| TCGA-EB-A5SG | 0 | 0 | 0 |  | 1 |  | 0 | 68.25 | 1 | 45.11 |
| TCGA-EB-A5SH | 1 | 1 | 0 |  | 1 | 3 | 0 | 54.02 | 1 | 35.01 |
| TCGA-EB-A5UL | 0 | 0 | 0 |  | 0 | 3 | 0 | 29.29 | 0 | 29.29 |
| TCGA-EB-A5UM | 0 | 0 | 1 |  | 1 | 2 | 0 | 25.61 | 0 | 25.61 |
| TCGA-EB-A5UN | 0 | 0 | 0 |  | 0 | 2 | 0 | 58.91 | 1 | 55.53 |
| TCGA-EB-A5VU | 1 | 0 | 0 |  | 0 | 3 | 1 | 10.55 | 1 | 7.13 |
| TCGA-EB-A5VV | 0 | 0 | 0 |  | 1 | 3 | 0 | 7.04 | 0 | 7.04 |
| TCGA-EB-A6L9 | 0 | 0 | 0 |  | 0 | 3 | 0 | 36.46 | 0 | 36.46 |
| TCGA-EB-A6QY | 0 | 0 | 0 |  | 0 | 2 | 0 | 12.56 | 1 | 1.18 |
| TCGA-EB-A6QZ | 0 | 0 | 0 |  | 1 | 2 | 1 | 11.57 | 1 | 4.24 |
| TCGA-EB-A6R0 | 0 | 0 | 0 |  | 1 | 2 | 1 | 19.99 | 0 | 19.99 |
| TCGA-EE-A17X | 1 | 1 | 0 |  | 0 | 1 | 1 | 29.82 | 1 | 17.46 |
| TCGA-EE-A17Y | 0 | 0 | 0 |  | 0 | 3 | 1 | 27.22 | 1 | 10.42 |
| TCGA-EE-A17Z | 0 | 0 | 0 |  | 0 | 2 | 1 | 8.65 | 1 | 4.54 |
| TCGA-EE-A180 | 0 | 0 | 0 |  | 0 | 3 | 1 | 94.98 | 1 | 27.94 |
| TCGA-EE-A181 | 0 | 0 | 0 |  | 1 | 2 | 1 | 33.73 | 1 | 25.91 |
| TCGA-EE-A182 | 1 | 0 | 0 |  | 1 | 3 | 1 | 14.7 | 1 | 8.88 |
| TCGA-EE-A184 | 0 | 0 | 0 |  | 0 | 1 | 1 | 68.15 | 1 | 43.04 |
| TCGA-EE-A185 | 0 | 0 | 0 |  | 1 | 3 | 1 | 4.96 | 1 | 2.79 |
| TCGA-EE-A20B | 0 | 0 | 0 |  | 1 | 2 | 0 | 133.81 | 0 | 133.81 |
| TCGA-EE-A20F | 1 | 0 | 0 |  | 0 | 1 | 0 | 91.56 | 0 | 91.56 |
| TCGA-EE-A20H | 0 | 1 | 0 |  | 0 | 1 | 1 | 168.26 | 1 | 153.43 |
| TCGA-EE-A20I | 0 | 0 | 0 |  | 0 | 4 | 1 | 13.55 | 1 | 1.74 |
| TCGA-EE-A29A | 0 | 0 | 0 |  | 0 | 3 | 1 | 63.35 | 1 | 28.5 |
| TCGA-EE-A29B | 0 | 0 | 0 |  | 0 | 2 | 1 | 85.08 | 1 | 16.11 |
| TCGA-EE-A29C | 0 | 0 | 0 |  | 0 | 1 | 1 | 78.97 | 1 | 6.71 |
| TCGA-EE-A29D | 0 | 0 | 0 |  | 0 | 3 | 1 | 13.97 | 0 | 13.97 |
| TCGA-EE-A29E | 0 | 0 | 1 |  | 0 | 3 | 0 | 63.78 | 0 | 63.78 |
| TCGA-EE-A29G | 0 | 0 | 0 |  | 0 | 3 | 1 | 72.06 | 1 | 23.87 |
| TCGA-EE-A29H | 0 | 0 | 0 |  | 1 | 1 | 0 | 64.63 | 1 | 40.47 |
| TCGA-EE-A29L | 1 | 0 | 0 |  | 0 | 3 | 1 | 2.6 | 1 | 1.81 |
| TCGA-EE-A29M | 0 | 0 | 0 |  | 1 | 1 | 0 | 56.84 | 0 | 56.84 |
| TCGA-EE-A29N | 0 | 0 | 0 |  | 0 | 2 | 1 | 18.61 | 1 | 15.71 |
| TCGA-EE-A29P | 0 | 0 | 0 |  | 1 | 2 | 0 | 56.42 | 1 | 54.84 |
| TCGA-EE-A29Q | 1 | 1 | 0 |  | 1 | 2 | 1 | 66.74 | 1 | 41.29 |
| TCGA-EE-A29R | 1 | 0 | 0 |  | 1 | 3 | 0 | 14.47 | 1 | 14.01 |
| TCGA-EE-A29S | 0 | 1 | 0 |  | 0 | 2 | 1 | 61.28 | 1 | 46.62 |
| TCGA-EE-A29T | 0 | 0 | 0 |  | 1 | *N/A* | 0 | 369.92 | 1 | 246.01 |
| TCGA-EE-A29V | 0 | 0 | 0 |  | 0 | 3 | 1 | 25.87 | 1 | 3.16 |
| TCGA-EE-A29X | 0 | 0 | 0 |  | 1 | 1 | 1 | 17.92 | 1 | 12.85 |
| TCGA-EE-A2A0 | 0 | 0 | 0 |  | 1 | 2 | 1 | 46.82 | 1 | 41 |
| TCGA-EE-A2A1 | 0 | 0 | 0 |  | 0 | 1 | 0 | 115.95 | 1 | 114.67 |
| TCGA-EE-A2A2 | 0 | 0 | 0 |  | 0 | 3 | 0 | 59.64 | 0 | 59.64 |
| TCGA-EE-A2A5 | 1 | 1 | 0 |  | 0 | 1 | 1 | 39.29 | 1 | 33.8 |
| TCGA-EE-A2A6 | 0 | 0 | 0 |  | 0 | 1 | 0 | 86.14 | 1 | 49.48 |
| TCGA-EE-A2GB | 0 | 0 | 0 |  | 0 | 3 | 0 | 59.28 | 0 | 59.28 |
| TCGA-EE-A2GC | 0 | 0 | 0 |  | 0 | 2 | 0 | 67.43 | 0 | 67.43 |
| TCGA-EE-A2GD | 0 | 0 | 0 |  | 1 | 2 | 1 | 340.14 | 1 | 121.28 |
| TCGA-EE-A2GE | 0 | 0 | 0 |  | 0 | 1 | 0 | 173.78 | 1 | 173.46 |
| TCGA-EE-A2GH | 0 | 0 | 0 |  | 0 | 1 | 0 | 220.24 | 0 | 220.24 |
| TCGA-EE-A2GI | 0 | 0 | 0 |  | 0 | 1 | 0 | 48.72 | 0 | 48.72 |
| TCGA-EE-A2GJ | 0 | 0 | 0 |  | 0 | 1 | 1 | 107.37 | 1 | 24.1 |
| TCGA-EE-A2GK | 0 | 0 | 0 |  | 1 | 1 | 0 | 54.74 | 1 | 49.25 |
| TCGA-EE-A2GL | 0 | 0 | 0 |  | 1 | 2 | 0 | 79.66 | 1 | 76.57 |
| TCGA-EE-A2GM | 1 | 0 | 0 |  | 1 | 2 | 0 | 75.48 | 1 | 40.04 |
| TCGA-EE-A2GN | 0 | 0 | 0 |  | 0 | 2 | 1 | 102.11 | 1 | 79.63 |
| TCGA-EE-A2GO | 0 | 0 | 0 |  | 1 | 2 | 0 | 126.8 | 1 | 72.85 |
| TCGA-EE-A2GP | 0 | 0 | 0 |  | 0 | 3 | 1 | 13.91 | 1 | 8.25 |
| TCGA-EE-A2GR | 0 | 0 | 0 |  | 0 | 2 | 1 | 42.77 | 1 | 42.77 |
| TCGA-EE-A2GS | 0 | 0 | 0 |  | 1 | 1 | 1 | 81.2 | 1 | 51.45 |
| TCGA-EE-A2GT | 0 | 0 | 0 |  | 0 | 2 | 0 | 44.88 | 1 | 9.24 |
| TCGA-EE-A2GU | 0 | 1 | 0 |  | 1 | 1 | 0 | 94.82 | 0 | 94.82 |
| TCGA-EE-A2M5 | 0 | 0 | 0 |  | 0 | 1 | 1 | 21.67 | 1 | 17.65 |
| TCGA-EE-A2M6 | 0 | 0 | 0 |  | 0 | 1 | 0 | 129.27 | 0 | 129.27 |
| TCGA-EE-A2M7 | 0 | 0 | 0 |  | 0 | 2 | 1 | 28.83 | 1 | 26.27 |
| TCGA-EE-A2M8 | 0 | 0 | 0 |  | 1 | 3 | 1 | 19.76 | 1 | 16.37 |
| TCGA-EE-A2MC | 0 | 0 | 0 |  | 0 | 1 | 1 | 61.51 | 1 | 29.46 |
| TCGA-EE-A2MD | 1 | 0 | 0 |  | 0 | 2 | 1 | 47.28 | 1 | 44.19 |
| TCGA-EE-A2ME | 0 | 0 | 0 |  | 0 | 1 | 1 | 103.26 | 1 | 88.86 |
| TCGA-EE-A2MF | 0 | 0 | 0 |  | 1 | 1 | 1 | 268.73 | 1 | 265.35 |
| TCGA-EE-A2MG | 0 | 0 | 0 |  | 0 | 1 | 1 | 103.2 | 1 | 84.99 |
| TCGA-EE-A2MH | 0 | 0 | 0 |  | 0 | 3 | 1 | 16.96 | 1 | 14.47 |
| TCGA-EE-A2MI | 0 | 0 | 0 |  | 0 | 2 | 1 | 204.66 | 1 | 87.88 |
| TCGA-EE-A2MJ | 0 | 0 | 0 |  | 0 | 3 | 1 | 96.23 | 1 | 13.02 |
| TCGA-EE-A2MK | 0 | 0 | 0 |  | 1 | 3 | 0 | 180.39 | 0 | 180.39 |
| TCGA-EE-A2ML | 0 | 0 | 0 |  | 0 | 2 | 1 | 216.66 | 1 | 163.82 |
| TCGA-EE-A2MM | 0 | 0 | 0 |  | 1 | 1 | 1 | 167.9 | 1 | 44.61 |
| TCGA-EE-A2MN | 1 | 0 | 0 |  | 0 | 1 | 1 | 47.54 | 1 | 31.86 |
| TCGA-EE-A2MP | 0 | 0 | 0 |  | 1 | 1 | 0 | 248.64 | 1 | 12 |
| TCGA-EE-A2MQ | 0 | 0 | 0 |  | 1 | 3 | 1 | 43.23 | 1 | 32.42 |
| TCGA-EE-A2MR | 0 | 0 | 0 |  | 0 | 1 | 0 | 134.4 | 1 | 103.1 |
| TCGA-EE-A2MS | 0 | 0 | 0 |  | 0 | 2 | 0 | 162.47 | 1 | 61.91 |
| TCGA-EE-A2MT | 0 | 0 | 0 |  | 0 | 1 | 0 | 71.21 | 0 | 71.21 |
| TCGA-EE-A2MU | 0 | 0 | 0 |  | 0 | 1 | 0 | 53.26 | 0 | 53.26 |
| TCGA-EE-A3AA | 0 | 0 | 0 |  | 0 | 3 | 0 | 124.31 | 0 | 124.31 |
| TCGA-EE-A3AB | 0 | 0 | 0 |  | 0 | 3 | 0 | 122.73 | 0 | 122.73 |
| TCGA-EE-A3AC | 0 | 0 | 0 |  | 0 | 3 | 0 | 64.04 | 0 | 64.04 |
| TCGA-EE-A3AD | 0 | 0 | 0 |  | 0 | 3 | 1 | 28.77 | 1 | 18.21 |
| TCGA-EE-A3AE | 1 | 0 | 0 |  | 1 | 1 | 0 | 54.51 | 0 | 54.51 |
| TCGA-EE-A3AF | 0 | 0 | 0 |  | 1 | 3 | 1 | 13.81 | 1 | 6.61 |
| TCGA-EE-A3AG | 0 | 0 | 0 |  | 0 | 3 | 1 | 41.59 | 1 | 7.53 |
| TCGA-EE-A3AH | 0 | 0 | 0 |  | 0 | 2 | 1 | 138.8 | 1 | 65.13 |
| TCGA-EE-A3J3 | 0 | 0 | 0 |  | 0 | 1 | 1 | 172.17 | 1 | 148.7 |
| TCGA-EE-A3J4 | 0 | 0 | 0 |  | 0 | 2 | 1 | 127.2 | 1 | 46.98 |
| TCGA-EE-A3J5 | 1 | 0 | 0 |  | 0 | 3 | 1 | 36.95 | 1 | 15.35 |
| TCGA-EE-A3J7 | 0 | 0 | 0 |  | 0 | 1 | 0 | 64.08 | 1 | 41.75 |
| TCGA-EE-A3J8 | 0 | 0 | 0 |  | 0 | 3 | 1 | 34.32 | 1 | 34.03 |
| TCGA-EE-A3JA | 0 | 0 | 0 |  | 0 | 1 | 1 | 53.19 | 1 | 17.72 |
| TCGA-EE-A3JB | 0 | 0 | 0 |  | 1 | 3 | 0 | 201.8 | 1 | 47.14 |
| TCGA-EE-A3JD | 0 | 0 | 0 |  | 0 | 3 | 1 | 27.35 | 1 | 11.97 |
| TCGA-EE-A3JE | 0 | 0 | 0 |  | 0 | 3 | 0 | 51.35 | 1 | 6.38 |
| TCGA-EE-A3JH | 0 | 0 | 0 |  | 0 | 1 | 0 | 134.33 | 1 | 17.65 |
| TCGA-EE-A3JI | 0 | 0 | 0 |  | 0 | 1 | 1 | 152.81 | 1 | 23.84 |
| TCGA-ER-A193 | 0 | 1 | 0 |  | 0 | 2 | 1 | 31.4 | 1 | 26.5 |
| TCGA-ER-A194 | 0 | 0 | 0 |  | 0 | *N/A* | 1 | 44.51 | 1 | 44.51 |
| TCGA-ER-A195 | 0 | 0 | 0 |  | 0 | *N/A* | 1 | 35.44 | 1 | 35.44 |
| TCGA-ER-A196 | 0 | 0 | 0 |  | 1 | 2 | 0 | 58.68 | 0 | 58.68 |
| TCGA-ER-A197 | 0 | 0 | 0 |  | 1 | 3 | 1 | 13.94 | 1 | 3.62 |
| TCGA-ER-A198 | 0 | 0 | 0 |  | 0 | *N/A* | 1 | 50.76 | *N/A* | *N/A* |
| TCGA-ER-A199 | 0 | 0 | 0 |  | 1 | 3 | 1 | 9.17 | 1 | 3.91 |
| TCGA-ER-A19A | 0 | 0 | 0 |  | 0 | 4 | 0 | 77.75 | 1 | 53.16 |
| TCGA-ER-A19B | 0 | 0 | 0 |  | 0 | *N/A* | 1 | 98.4 | 1 | 27.25 |
| TCGA-ER-A19C | 0 | 0 | 0 |  | 0 | 1 | 1 | 48.89 | 1 | 48.89 |
| TCGA-ER-A19D | 0 | 0 | 0 |  | 1 | 1 | 1 | 12.59 | 1 | 11.74 |
| TCGA-ER-A19E | 0 | 0 | 0 |  | 1 | 1 | 1 | 13.02 | 1 | 2.1 |
| TCGA-ER-A19F | 0 | 0 | 0 |  | 0 | *N/A* | 1 | 26.37 | 0 | 26.37 |
| TCGA-ER-A19G | 0 | 0 | 0 |  | 1 | *N/A* | 0 | 302.07 | 0 | 302.07 |
| TCGA-ER-A19H | 0 | 0 | 0 |  | 0 | *N/A* | 1 | 152.35 | 0 | 152.35 |
| TCGA-ER-A19J | 0 | 0 | 0 |  | 0 | 4 | 1 | 6.44 | 1 | 3.22 |
| TCGA-ER-A19K | 0 | 0 | 1 |  | 1 | 2 | 1 | 15.42 | 1 | 6.94 |
| TCGA-ER-A19L | 0 | 0 | 1 |  | 0 | *N/A* | 1 | 131.51 | 1 | 121.28 |
| TCGA-ER-A19M | 0 | 0 | 0 |  | 0 | 1 | 1 | 61.05 | 1 | 37.87 |
| TCGA-ER-A19N | 0 | 0 | 0 |  | 0 | *N/A* | 1 | 44.09 | *N/A* | *N/A* |
| TCGA-ER-A19O | 0 | 0 | 0 |  | 0 | 3 | *N/A* | *N/A* | 1 | 10.13 |
| TCGA-ER-A19P | 0 | 0 | 0 |  | 1 | *N/A* | 1 | 162.08 | 1 | 143.08 |
| TCGA-ER-A19Q | 0 | 0 | 0 |  | 1 | *N/A* | 1 | 50.89 | 1 | 41.39 |
| TCGA-ER-A19S | 0 | 0 | 0 |  | 1 | *N/A* | 0 | 49.48 | 0 | 49.48 |
| TCGA-ER-A19T | 1 | 0 | 0 |  | 0 | 4 | 1 | 8.88 | 1 | 3.88 |
| TCGA-ER-A19W | 0 | 0 | 0 |  | 1 | *N/A* | 1 | 148.17 | 1 | 135.65 |
| TCGA-ER-A1A1 | 0 | 0 | 0 |  | 0 | 3 | 0 | 105.07 | 1 | 64.8 |
| TCGA-ER-A2NB | 0 | 0 | 0 |  | 0 | 3 | 1 | 28.18 | 1 | 15.16 |
| TCGA-ER-A2NC | 0 | 0 | 0 |  | 0 | 1 | 1 | 43.82 | 1 | 38.04 |
| TCGA-ER-A2ND | 0 | 0 | 0 |  | 1 | 3 | 1 | 23.34 | 1 | 23.34 |
| TCGA-ER-A2NF | 0 | 0 | 0 |  | 0 | 3 | 1 | 28.83 | 1 | 7.56 |
| TCGA-ER-A2NG | 0 | 0 | 0 |  | 1 | 3 | 1 | 48.99 | 1 | 25.18 |
| TCGA-ER-A2NH | 0 | 0 | 0 |  | 0 | 3 | 0 | 41.56 | 0 | 41.56 |
| TCGA-ER-A3ES | 0 | 0 | 0 |  | 0 | *N/A* | 1 | 247.03 | 1 | 247.03 |
| TCGA-ER-A3ET | 0 | 0 | 1 |  | 1 | 3 | 1 | 93.01 | 1 | 80.05 |
| TCGA-ER-A3EV | 0 | 0 | 0 |  | 0 | 3 | 1 | 46.98 | 1 | 23.93 |
| TCGA-ER-A3PL | 0 | 0 | 1 |  | 0 | 4 | 0 | 33.21 | 1 | 31.07 |
| TCGA-ER-A42H | 0 | 0 | 0 |  | 0 | *N/A* | 1 | 14.01 | 1 | 8.51 |
| TCGA-ER-A42K | 0 | 0 | 0 |  | 1 | 3 | 1 | 12.95 | 1 | 6.77 |
| TCGA-ER-A42L | 0 | 0 | 0 |  | 0 | 2 | 0 | 149.03 | 1 | 125.32 |
| TCGA-FR-A2OS | 0 | 0 | 0 |  | 1 | 2 | 1 | 12.1 | 1 | 9.3 |
| TCGA-FR-A3R1 | 0 | 0 | 0 |  | 0 | 2 | 0 | 22.52 | 1 | 22.29 |
| TCGA-FR-A3YN | 1 | 0 | 0 |  | 0 | 1 | 0 | 92.97 | 0 | 92.97 |
| TCGA-FR-A44A | 0 | 0 | 0 |  | 1 | 2 | 0 | 174.21 | 0 | 174.21 |
| TCGA-FR-A69P | 1 | 0 | 0 |  | 1 | 3 | 0 | 15.71 | 1 | 9.01 |
| TCGA-FR-A726 | 0 | 0 | 0 |  | 0 | 2 | 1 | 10.03 | 1 | 8.98 |
| TCGA-FR-A728 | 0 | 0 | 0 |  | 1 | 3 | 0 | 19.17 | 1 | 2.1 |
| TCGA-FR-A729 | 0 | 0 | 0 |  | 1 | 1 | 0 | 220.8 | 1 | 207.09 |
| TCGA-FR-A7U8 | 0 | 0 | 0 |  | 0 | 3 | 0 | 27.85 | 1 | 18.64 |
| TCGA-FR-A7U9 | 0 | 0 | 0 |  | 1 | 3 | 0 | 18.77 | 1 | 3.12 |
| TCGA-FR-A7UA | 0 | 0 | 0 |  | 1 | 1 | 0 | 38.27 | 0 | 38.27 |
| TCGA-FR-A8YC | 0 | 0 | 0 |  | 0 | 2 | 1 | 34.82 | 1 | 34.82 |
| TCGA-FR-A8YD | 0 | 0 | 0 |  | 1 | 2 | 1 | 36.26 | 1 | 36.26 |
| TCGA-FR-A8YE | 0 | 0 | 0 |  | 0 | 1 | 0 | 104.42 | 0 | 104.42 |
| TCGA-FS-A1YW | 0 | 0 | 0 |  | 0 | 1 | 1 | 216.92 | 1 | 216.92 |
| TCGA-FS-A1YX | 0 | 1 | 0 |  | 1 | 1 | 1 | 48.59 | 1 | 48.59 |
| TCGA-FS-A1YY | 0 | 0 | 0 |  | 1 | 2 | 1 | 228.59 | 1 | 222.8 |
| TCGA-FS-A1Z0 | 0 | 0 | 0 |  | 1 | 1 | 1 | 202.65 | 1 | 180.92 |
| TCGA-FS-A1Z3 | 0 | 0 | 0 |  | 1 | 4 | 1 | 20.91 | 1 | 9.07 |
| TCGA-FS-A1Z4 | 0 | 0 | 0 |  | 0 | 1 | 1 | 28.08 | 1 | 28.08 |
| TCGA-FS-A1Z7 | 1 | 0 | 0 |  | 0 | 3 | 1 | 7.79 | 1 | 4.47 |
| TCGA-FS-A1ZA | 1 | 0 | 0 |  | 1 | 3 | 1 | 27.71 | 1 | 21.8 |
| TCGA-FS-A1ZB | 0 | 0 | 0 |  | 0 | 2 | 1 | 48.85 | 1 | 48.03 |
| TCGA-FS-A1ZC | 0 | 0 | 0 |  | 0 | 2 | 1 | 357.37 | 1 | 339.74 |
| TCGA-FS-A1ZD | 0 | 0 | 0 |  | 0 | 2 | 1 | 53.52 | 1 | 49.22 |
| TCGA-FS-A1ZE | 0 | 0 | 0 |  | 0 | 2 | 1 | 46.45 | 1 | 25.02 |
| TCGA-FS-A1ZF | 0 | 0 | 0 |  | 1 | 2 | 1 | 15.45 | 1 | 15.45 |
| TCGA-FS-A1ZG | 0 | 0 | 0 |  | 1 | 3 | 1 | 9.7 | 1 | 4.6 |
| TCGA-FS-A1ZH | 0 | 0 | 0 |  | 1 | 4 | 1 | 32.74 | 1 | 18.97 |
| TCGA-FS-A1ZJ | 0 | 0 | 0 |  | 1 | 1 | 1 | 47.37 | 1 | 40.27 |
| TCGA-FS-A1ZK | 1 | 0 | 0 |  | 0 | 2 | 1 | 23.93 | 1 | 12.07 |
| TCGA-FS-A1ZM | 0 | 0 | 0 |  | 0 | 3 | 0 | 101.26 | 0 | 101.26 |
| TCGA-FS-A1ZN | 0 | 0 | 0 |  | 0 | 3 | 1 | 24 | 1 | 11.87 |
| TCGA-FS-A1ZP | 0 | 0 | 0 |  | 0 | 2 | 1 | 74.73 | 1 | 74.73 |
| TCGA-FS-A1ZQ | 0 | 0 | 0 |  | 0 | 2 | 1 | 133.54 | 1 | 129.53 |
| TCGA-FS-A1ZR | 0 | 0 | 0 |  | 0 | 2 | 1 | 11.41 | 1 | 5 |
| TCGA-FS-A1ZS | 0 | 0 | 0 |  | 0 | 1 | 0 | 148.8 | 1 | 94.06 |
| TCGA-FS-A1ZT | 0 | 0 | 0 |  | 0 | 3 | 0 | 53.16 | 0 | 53.16 |
| TCGA-FS-A1ZU | 1 | 0 | 0 |  | 1 | 2 | 1 | 26.56 | 1 | 22.32 |
| TCGA-FS-A1ZW | 0 | 0 | 0 |  | 0 | 3 | 0 | 49.48 | 0 | 49.48 |
| TCGA-FS-A1ZY | 0 | 0 | 0 |  | 0 | 2 | 1 | 27.09 | 1 | 27.09 |
| TCGA-FS-A1ZZ | 0 | 0 | 0 |  | 1 | 2 | 1 | 27.02 | 1 | 27.02 |
| TCGA-FS-A4F0 | 0 | 0 | 0 |  | 1 | 2 | 0 | 77.82 | 1 | 28.67 |
| TCGA-FS-A4F2 | 0 | 1 | 0 |  | 1 | 2 | 1 | 50.14 | 1 | 50.14 |
| TCGA-FS-A4F4 | 1 | 0 | 1 |  | 0 | 2 | 1 | 66.67 | 1 | 66.67 |
| TCGA-FS-A4F5 | 1 | 1 | 0 |  | 1 | 1 | 1 | 28.73 | 1 | 28.73 |
| TCGA-FS-A4F8 | 0 | 0 | 0 |  | 0 | 1 | 1 | 174.84 | 1 | 174.84 |
| TCGA-FS-A4F9 | 0 | 1 | 0 |  | 0 | 3 | 0 | 34.03 | 1 | 12.36 |
| TCGA-FS-A4FB | 0 | 0 | 0 |  | 1 | 3 | 1 | 26.73 | 1 | 26.73 |
| TCGA-FS-A4FC | 0 | 0 | 0 |  | 1 | 2 | 1 | 54.41 | 1 | 54.41 |
| TCGA-FS-A4FD | 0 | 0 | 0 |  | 0 | 3 | 1 | 80.68 | 1 | 66.08 |
| TCGA-FW-A3I3 | 1 | 0 | 0 |  | 1 | 4 | 0 | 17.46 | 0 | 17.46 |
| TCGA-FW-A3R5 | 0 | 0 | 0 |  | 0 | 3 | 0 | 36.95 | 0 | 36.95 |
| TCGA-FW-A3TU | 0 | 1 | 0 |  | 1 | *N/A* | 1 | 55.59 | 1 | 55.59 |
| TCGA-FW-A3TV | 0 | 0 | 0 |  | 1 | 3 | 0 | 13.51 | 1 | 4.57 |
| TCGA-FW-A5DX | 1 | 0 | 0 |  | 0 | 3 | 0 | 21.04 | 0 | 21.04 |
| TCGA-FW-A5DY | 0 | 0 | 0 |  | 1 | 3 | 0 | 19.3 | 0 | 19.3 |
| TCGA-GF-A2C7 | 1 | 0 | 1 |  | 0 | 2 | 0 | 0.69 | 0 | 0.69 |
| TCGA-GF-A3OT | 0 | 0 | 0 |  | 1 | 3 | 0 | 9.9 | 1 | 8.15 |
| TCGA-GF-A4EO | 0 | 0 | 0 |  | 1 | 3 | 0 | 19.43 | 0 | 19.43 |
| TCGA-GF-A6C8 | 0 | 0 | 0 |  | 1 | 2 | 0 | 2.04 | 0 | 2.04 |
| TCGA-GF-A6C9 | 0 | 0 | 0 |  | 0 | 3 | 0 | 15.78 | 0 | 15.78 |
| TCGA-GF-A769 | 0 | 0 | 0 |  | 0 | 2 | 1 | 35.18 | 1 | 35.18 |
| TCGA-GN-A262 | 0 | 0 | 0 |  | 1 | *N/A* | 0 | 139.89 | 1 | 95.6 |
| TCGA-GN-A263 | 0 | 0 | 0 |  | 0 | 4 | 1 | 15.35 | 1 | 1.28 |
| TCGA-GN-A264 | 1 | 0 | 0 |  | 0 | *N/A* | 1 | 117.93 | 1 | 105.89 |
| TCGA-GN-A265 | 0 | 0 | 0 |  | 0 | *N/A* | 0 | 96.92 | 1 | 3.25 |
| TCGA-GN-A266 | 0 | 0 | 0 |  | 0 | *N/A* | 1 | 10.13 | 1 | 3.58 |
| TCGA-GN-A267 | 1 | 1 | 0 |  | 0 | 3 | 1 | 64.44 | 1 | 14.99 |
| TCGA-GN-A268 | 0 | 1 | 0 |  | 1 | 2 | 1 | 62.79 | 1 | 29.85 |
| TCGA-GN-A269 | 0 | 0 | 0 |  | 0 | 3 | 1 | 5.59 | 1 | 5.59 |
| TCGA-GN-A26A | 0 | 0 | 0 |  | 1 | 3 | 1 | 32.48 | 1 | 24.66 |
| TCGA-GN-A26C | 0 | 0 | 0 |  | 0 | 3 | 1 | 26.99 | 1 | 19.5 |
| TCGA-GN-A26D | 1 | 0 | 0 |  | 1 | 2 | 1 | 48 | 1 | 20.45 |
| TCGA-GN-A4U3 | 0 | 1 | 0 |  | 0 | 3 | 0 | 121.91 | 1 | 74.76 |
| TCGA-GN-A4U4 | 0 | 0 | 0 |  | 0 | 2 | 0 | 39.35 | 1 | 7.13 |
| TCGA-GN-A4U5 | 0 | 0 | 0 |  | 1 | 1 | 0 | 38.01 | 0 | 38.01 |
| TCGA-GN-A4U7 | 0 | 0 | 0 |  | 1 | 3 | 1 | 10.42 | 1 | 6.02 |
| TCGA-GN-A4U8 | 0 | 0 | 0 |  | 0 | *N/A* | 0 | 48.89 | 0 | 48.89 |
| TCGA-GN-A4U9 | 1 | 0 | 0 |  | 0 | 3 | 1 | 22.13 | 1 | 4.96 |
| TCGA-GN-A8LK | 0 | 0 | 0 |  | 0 | 1 | 1 | 50.1 | 1 | 14.33 |
| TCGA-GN-A8LL | 1 | 1 | 0 |  | 1 | 2 | 1 | 21.37 | 1 | 21.37 |
| TCGA-GN-A9SD | 0 | 0 | 0 |  | 1 | 1 | 1 | 59.41 | 1 | 37.38 |
| TCGA-HR-A2OG | 0 | 0 | 0 |  | 1 | *N/A* | 0 | 0.23 | 0 | 0.23 |
| TCGA-HR-A2OH | 0 | 0 | 0 |  | 1 | 3 | 1 | 65.88 | 1 | 65.88 |
| TCGA-IH-A3EA | 0 | 0 | 0 |  | 0 | 2 | 0 | 17.23 | 1 | 10.32 |
| TCGA-LH-A9QB | 0 | 1 | 0 |  | 1 | *N/A* | 0 | 368.77 | 0 | 368.77 |
| TCGA-OD-A75X | 0 | 0 | 0 |  | 0 | *N/A* | 1 | 297.89 | 1 | 295.46 |
| TCGA-QB-A6FS | 0 | 0 | 0 |  | 0 | 3 | 0 | 7.23 | 1 | 2.07 |
| TCGA-QB-AA9O | 0 | 0 | 0 |  | 0 | 3 | 1 | 18.05 | 1 | 18.05 |
| TCGA-RP-A690 | 0 | 0 | 0 |  | 1 | *N/A* | 0 | 0.2 | 0 | 0.2 |
| TCGA-RP-A693 | 0 | 0 | 0 |  | 0 | 4 | 0 | 0.33 | 0 | 0.33 |
| TCGA-RP-A694 | 0 | 0 | 0 |  | 0 | 4 | 0 | 0.69 | 0 | 0.69 |
| TCGA-W3-A824 | 0 | 0 | 0 |  | 0 | 1 | 0 | 228.16 | 1 | 79.1 |
| TCGA-W3-A825 | 0 | 0 | 0 |  | 1 | 2 | 1 | 63.02 | 1 | 63.02 |
| TCGA-W3-A828 | 0 | 0 | 0 |  | 0 | 2 | 1 | 121.08 | 0 | 121.08 |
| TCGA-W3-AA1O | 0 | 0 | 0 |  | 0 | 3 | 1 | 4.01 | 1 | 4.01 |
| TCGA-W3-AA1Q | 0 | 0 | 0 |  | 0 | 3 | 1 | 69.07 | 1 | 35.05 |
| TCGA-W3-AA1R | 0 | 0 | 0 |  | 0 | 2 | 1 | 111.09 | 1 | 34.09 |
| TCGA-W3-AA1V | 0 | 0 | 0 |  | 0 | 2 | 1 | 42.08 | 1 | 40.08 |
| TCGA-W3-AA1W | 0 | 0 | 0 |  | 0 | 2 | 0 | 219.15 | 1 | 11.01 |
| TCGA-W3-AA21 | 0 | 0 | 0 |  | 0 | 1 | 1 | 105.04 | 1 | 97.05 |
| TCGA-WE-A8JZ | 1 | 0 | 1 |  | 0 | 3 | 0 | 24.03 | 0 | 24.03 |
| TCGA-WE-A8K1 | 0 | 0 | 0 |  | 0 | 3 | 0 | 49.05 | 0 | 49.05 |
| TCGA-WE-A8K5 | 0 | 0 | 0 |  | 0 | 4 | 1 | 61.15 | 1 | 49.25 |
| TCGA-WE-A8K6 | 0 | 0 | 0 |  | 0 | 3 | 0 | 17.95 | 1 | 8.42 |
| TCGA-WE-A8ZM | 0 | 0 | 0 |  | 0 | 3 | 0 | 101.32 | 1 | 21.24 |
| TCGA-WE-A8ZN | 0 | 0 | 0 |  | 0 | 2 | 0 | 58.98 | 1 | 47.64 |
| TCGA-WE-A8ZO | 1 | 0 | 0 |  | 1 | 3 | 0 | 70.52 | 1 | 45.07 |
| TCGA-WE-A8ZQ | 0 | 1 | 0 |  | 0 | 2 | 0 | 63.22 | 1 | 57.76 |
| TCGA-WE-A8ZT | 0 | 0 | 0 |  | 1 | 4 | 0 | 11.8 | 1 | 2.27 |
| TCGA-WE-A8ZX | 0 | 1 | 0 |  | 0 | 3 | 0 | 35.8 | 1 | 10.72 |
| TCGA-WE-A8ZY | 1 | 0 | 0 |  | 0 | 2 | 1 | 49.51 | 1 | 45.67 |
| TCGA-WE-AA9Y | 0 | 0 | 0 |  | 0 | 3 | 0 | 12.16 | 0 | 12.16 |
| TCGA-WE-AAA0 | 0 | 0 | 0 |  | 0 | 1 | 0 | 40.41 | 1 | 21.37 |
| TCGA-WE-AAA3 | 0 | 0 | 0 |  | 1 | 3 | 0 | 21.4 | 0 | 21.4 |
| TCGA-WE-AAA4 | 0 | 0 | 0 |  | 1 | 3 | 0 | 24.99 | 1 | 17.19 |
| TCGA-XV-AB01 | 0 | 0 | 0 |  | 1 | 2 | 0 | 13.25 | 0 | 13.25 |
| TCGA-YD-A89C | 0 | 0 | 0 |  | 1 | 1 | 0 | 6.9 | 0 | 6.9 |
| TCGA-YD-A9TA | 0 | 0 | 0 |  | 0 |  | 0 | 49.18 | 0 | 49.18 |
| TCGA-YG-AA3O | 1 | 0 | 1 |  | 0 |  | 1 | 37.94 | 1 | 32.84 |
| TCGA-YG-AA3P | 1 | 0 | 0 |  | 1 | 2 | 0 | 14.43 | 0 | 14.43 |
| TCGA-Z2-A8RT | 1 | 0 | 0 |  | 1 | 2 | 0 | 27.58 | 0 | 27.58 |
| TCGA-Z2-AA3S | 0 | 0 | 0 |  | 0 | 1 | 0 | 96.99 | 1 | 89.65 |
| TCGA-Z2-AA3V | 0 | 0 | 0 |  | 1 | 1 | 0 | 15.98 | 0 | 15.98 |
| Pat02 | 1 | 0 | 0 |  | 1 | 4 | 0 | 54.4 | 1 | 17.93 |
| Pat03 | 0 | 0 | 0 |  | 1 | 4 | 1 | 3.33 | 1 | 2.53 |
| Pat04 | 0 | 0 | 0 |  | 0 | 4 | 0 | 32.9 | 0 | 21.53 |
| Pat06 | 0 | 0 | 0 |  | 0 | 4 | 1 | 5.37 | 1 | 2.57 |
| Pat07 | 1 | 0 | 1 |  | 0 | 3 | 0 | 35 | 1 | 24.03 |
| Pat08 | 0 | 0 | 0 |  | 0 | 4 | 1 | 4.67 | 1 | 2.43 |
| Pat100 | 0 | 0 | 0 |  | 0 | 4 | 1 | 12 | 1 | 3.7 |
| Pat101 | 0 | 1 | 1 |  | 0 | 4 | 1 | 9.6 | 1 | 2.57 |
| Pat103 | 0 | 0 | 0 |  | 0 | 4 | 1 | 34.93 | 0 | 32.3 |
| Pat104 | 0 | 0 | 0 |  | 1 | 4 | 1 | 7.9 | 0 | 7.9 |
| Pat105 | 0 | 0 | 0 |  | 0 | 3 | 0 | 34.9 | 0 | 23 |
| Pat106 | 0 | 0 | 0 |  | 1 | 4 | 1 | 8.33 | 1 | 4.57 |
| Pat109 | 0 | 0 | 0 |  | 0 | 4 | 1 | 2.77 | 1 | 0.63 |
| Pat11 | 0 | 0 | 0 |  | 0 | 4 | 0 | 26.33 | 1 | 4.2 |
| Pat110 | 1 | 0 | 0 |  | 0 | 4 | 1 | 10.67 | 1 | 2.43 |
| Pat113 | 0 | 0 | 1 |  | 0 | 4 | 1 | 10.03 | 1 | 1.73 |
| Pat115 | 1 | 1 | 0 |  | 0 | 4 | 1 | 4.83 | 1 | 2.47 |
| Pat117 | 1 | 0 | 0 |  | 0 | 4 | 0 | 30.47 | 1 | 6.07 |
| Pat118 | 0 | 0 | 0 |  | 1 | 4 | 1 | 10.43 | 1 | 4 |
| Pat119 | 0 | 0 | 0 |  | 0 | 3 | 0 | 26.93 | 1 | 3.6 |
| Pat121 | 0 | 0 | 0 |  | 0 | 4 | 1 | 4.07 | 1 | 2.8 |
| Pat123 | 0 | 0 | 0 |  | 1 | 4 | 1 | 28.43 | 1 | 18.13 |
| Pat124 | 0 | 0 | 0 |  | 0 | 4 | 1 | 4.87 | 1 | 2.23 |
| Pat126 | 0 | 0 | 0 |  | 0 | 4 | 0 | 21.37 | 1 | 6.23 |
| Pat127 | 0 | 1 | 0 |  | 1 | 4 | 1 | 11.07 | 1 | 2.8 |
| Pat128 | 1 | 0 | 0 |  | 0 | 4 | 1 | 3.77 | 1 | 2.7 |
| Pat129 | 0 | 0 | 0 |  | 0 | 4 | 0 | 17.97 | 1 | 2.27 |
| Pat13 | 0 | 0 | 0 |  | 0 | 3 | 1 | 24.4 | 1 | 3.47 |
| Pat130 | 0 | 0 | 0 |  | 0 | 4 | 1 | 1.5 | 1 | 0.57 |
| Pat131 | 0 | 0 | 0 |  | 0 | 4 | 1 | 8.57 | 1 | 6.5 |
| Pat132 | 0 | 0 | 0 |  | 0 | 4 | 0 | 22.53 | 1 | 6.23 |
| Pat133 | 0 | 1 | 0 |  | 0 | 4 | 1 | 18.07 | 1 | 4.47 |
| Pat135 | 0 | 1 | 0 |  | 1 | 4 | 1 | 2.7 | 1 | 2.43 |
| Pat138 | 0 | 0 | 0 |  | 1 | 4 | 1 | 49.27 | 1 | 19.37 |
| Pat139 | 0 | 0 | 1 |  | 0 | 4 | 1 | 3.3 | 1 | 1.53 |
| Pat14 | 0 | 0 | 0 |  | 1 | 4 | 1 | 5.43 | 1 | 2.8 |
| Pat140 | 0 | 0 | 0 |  | 0 | 4 | 1 | 16.4 | 1 | 3.8 |
| Pat143 | 1 | 1 | 1 |  | 0 | 4 | 1 | 5.1 | 1 | 3 |
| Pat147 | 0 | 0 | 0 |  | 0 | 4 | 1 | 7.47 | 1 | 4 |
| Pat148 | 1 | 1 | 0 |  | 1 | 4 | 1 | 2.8 | 1 | 1.6 |
| Pat15 | 1 | 0 | 0 |  | 0 | 4 | 1 | 1.67 | 1 | 1.03 |
| Pat151 | 0 | 0 | 0 |  | 0 | 4 | 1 | 6.8 | 1 | 3.53 |
| Pat157 | 0 | 1 | 0 |  | 1 | 4 | 1 | 2.87 | 1 | 1.77 |
| Pat159 | 0 | 1 | 0 |  | 0 | 4 | 0 | 28.07 | 1 | 3.33 |
| Pat16 | 1 | 0 | 0 |  | 1 | 4 | 1 | 27.03 | 1 | 2.8 |
| Pat160 | 1 | 1 | 1 |  | 0 | 4 | 1 | 5.07 | 1 | 2.7 |
| Pat162 | 0 | 0 | 0 |  | 1 | 4 | 1 | 7.03 | 1 | 2.6 |
| Pat163 | 0 | 0 | 0 |  | 0 | 4 | 0 | 24.97 | 1 | 2.53 |
| Pat165 | 0 | 0 | 0 |  | 1 | 4 | 1 | 3.83 | 1 | 3.17 |
| Pat166 | 0 | 0 | 1 |  | 0 | 4 | 1 | 2.57 | 1 | 2.53 |
| Pat167 | 0 | 0 | 0 |  | 0 | 4 | 1 | 13.6 | 1 | 2.8 |
| Pat168 | 0 | 1 | 1 |  | 0 | 4 | 1 | 2.23 | 1 | 2.23 |
| Pat17 | 0 | 0 | 0 |  | 0 | 4 | 1 | 6.83 | 1 | 2.83 |
| Pat170 | 0 | 0 | 0 |  | 0 | 4 | 1 | 3.6 | 1 | 3.23 |
| Pat171 | 0 | 1 | 0 |  | 0 | 4 | 1 | 15.53 | 1 | 2.83 |
| Pat174 | 0 | 0 | 0 |  | 1 | 4 | 0 | 22.93 | 1 | 4.47 |
| Pat175 | 0 | 0 | 0 |  | 1 | 4 | 1 | 3 | 1 | 3 |
| Pat18 | 0 | 0 | 0 |  | 1 | 3 | 0 | 26.6 | 1 | 3.73 |
| Pat19 | 0 | 0 | 0 |  | 0 | 4 | 1 | 5.83 | 1 | 0.7 |
| Pat21 | 0 | 0 | 0 |  | 0 | 4 | 1 | 22.53 | 1 | 18.6 |
| Pat24 | 0 | 0 | 0 |  | 1 | 4 | 0 | 32.37 | 0 | 21.93 |
| Pat25 | 0 | 0 | 0 |  | 0 | 4 | 1 | 10.83 | 1 | 2.23 |
| Pat27 | 1 | 1 | 0 |  | 0 | 4 | 0 | 45.97 | 1 | 4.63 |
| Pat28 | 0 | 0 | 0 |  | 0 | 4 | 1 | 39.47 | 1 | 2.57 |
| Pat29 | 0 | 0 | 0 |  | 0 | 4 | 0 | 44.2 | 1 | 11.37 |
| Pat32 | 0 | 0 | 0 |  | 0 | 4 | 1 | 4.9 | 1 | 2.33 |
| Pat33 | 1 | 0 | 0 |  | 0 | 4 | 1 | 7.03 | 1 | 7.03 |
| Pat36 | 0 | 0 | 0 |  | 1 | 4 | 1 | 1.8 | 1 | 1.37 |
| Pat37 | 1 | 0 | 1 |  | 1 | 4 | 1 | 2.33 | 1 | 0.77 |
| Pat38 | 1 | 0 | 0 |  | 0 | 4 | 0 | 51.3 | 1 | 10.07 |
| Pat39 | 0 | 0 | 0 |  | 0 | 4 | 0 | 49.57 | 0 | 49.57 |
| Pat40 | 0 | 0 | 0 |  | 0 | 4 | 1 | 1.13 | 1 | 1.13 |
| Pat41 | 0 | 0 | 0 |  | 0 | 4 | 1 | 4.27 | 1 | 1.8 |
| Pat43 | 0 | 0 | 0 |  | 1 | 4 | 1 | 1.23 | 1 | 1.23 |
| Pat44 | 0 | 0 | 0 |  | 1 | 4 | 1 | 9.03 | 1 | 2.07 |
| Pat45 | 1 | 1 | 0 |  | 0 | 4 | 1 | 2.97 | 1 | 2.03 |
| Pat46 | 1 | 0 | 0 |  | 1 | 4 | 1 | 5.33 | 1 | 1.2 |
| Pat47 | 0 | 0 | 0 |  | 0 | 4 | 0 | 36.87 | 0 | 36.87 |
| Pat49 | 0 | 0 | 0 |  | 0 | 4 | 0 | 34.47 | 1 | 5.43 |
| Pat50 | 0 | 0 | 0 |  | 0 | 4 | 1 | 2.17 | 1 | 2.17 |
| Pat54 | 0 | 0 | 0 |  | 0 | 4 | 1 | 6.93 | 1 | 2.7 |
| Pat55 | 1 | 0 | 0 |  | 1 | 4 | 1 | 6.43 | 1 | 2.6 |
| Pat56 | 1 | 0 | 0 |  | 0 | 4 | 1 | 7.83 | 1 | 3.2 |
| Pat57 | 0 | 0 | 0 |  | 0 | 3 | 1 | 8.33 | 1 | 2.27 |
| Pat58 | 0 | 1 | 0 |  | 1 | 3 | 1 | 21.73 | 1 | 3.3 |
| Pat59 | 0 | 0 | 0 |  | 0 | 4 | 1 | 7.47 | 1 | 2.53 |
| Pat60 | 0 | 0 | 0 |  | 0 | 4 | 1 | 9.07 | 1 | 2.97 |
| Pat62 | 0 | 0 | 0 |  | 0 | 4 | 1 | 20.07 | 1 | 2.57 |
| Pat63 | 1 | 0 | 0 |  | 0 | 3 | 1 | 34.63 | 1 | 23.93 |
| Pat64 | 1 | 0 | 0 |  | 0 | 4 | 1 | 3.53 | 1 | 2.3 |
| Pat66 | 0 | 0 | 0 |  | 1 | 3 | 0 | 21.77 | 1 | 19.93 |
| Pat67 | 0 | 0 | 0 |  | 0 | 4 | 1 | 2.63 | 1 | 2.63 |
| Pat70 | 0 | 0 | 0 |  | 0 | 4 | 1 | 17.73 | 1 | 0.5 |
| Pat71 | 0 | 0 | 0 |  | 0 | 4 | 1 | 4.57 | 1 | 3.27 |
| Pat73 | 0 | 0 | 0 |  | 0 | 4 | 1 | 14.73 | 1 | 13.6 |
| Pat74 | 0 | 0 | 0 |  | 0 | 4 | 1 | 6.43 | 1 | 2.67 |
| Pat76 | 0 | 0 | 0 |  | 0 | 4 | 1 | 4.63 | 1 | 2.33 |
| Pat77 | 0 | 0 | 0 |  | 0 | 3 | 0 | 34.3 | 0 | 8.03 |
| Pat78 | 0 | 0 | 0 |  | 1 | 4 | 1 | 1.27 | 1 | 1.27 |
| Pat79 | 1 | 0 | 1 |  | 0 | 4 | 1 | 26.7 | 1 | 13.5 |
| Pat80 | 0 | 0 | 0 |  | 0 | 4 | 1 | 24.13 | 1 | 6.07 |
| Pat81 | 0 | 0 | 0 |  | 1 | 4 | 1 | 20.93 | 1 | 2.53 |
| Pat82 | 0 | 0 | 0 |  | 1 | 4 | 1 | 3.47 | 1 | 2.5 |
| Pat83 | 1 | 0 | 0 |  | 0 | 4 | 1 | 34.23 | 1 | 3.57 |
| Pat85 | 0 | 0 | 0 |  | 0 | 4 | 1 | 15.27 | 1 | 2.77 |
| Pat86 | 1 | 1 | 0 |  | 0 | 4 | 1 | 9.77 | 1 | 5.1 |
| Pat88 | 0 | 0 | 0 |  | 1 | 4 | 0 | 32.97 | 0 | 22.17 |
| Pat90 | 0 | 0 | 0 |  | 0 | 4 | 0 | 33.5 | 0 | 22.3 |
| Pat92 | 0 | 0 | 0 |  | 0 | 4 | 1 | 4.1 | 1 | 2.8 |
| Pat98 | 0 | 1 | 0 |  | 1 | 4 | 1 | 4.67 | 1 | 2.57 |

N/A: not applicable; OS: Overall Survival; DFS: Disease-free Survival; a. Samples were obtained from (Skin Cutaneous Melanoma (TCGA, PanCancer Atlas)^1^ and Metastatic Melanoma (DFCI, Science 2015) ^2^ with CNV and survival information; b. *MALAT1* CNV status (0: without *MALAT1* CNV deficiency, 1: with *MALAT1* CNV deficiency); c. *LINC00943* CNV status (0: without *LINC00943* CNV deficiency, 1: with *LINC00943* CNV deficiency); d. *LINC00261* CNV status (0: without *LINC00261* CNV deficiency, 1: with *LINC00261* CNV deficiency); e. age = 57.76±15.83; f. 0: male; 1: female; g. 1: stage 0/I, 2: stage II, 3: stage III, 4: stage IV; h. 0: alive/censored; 1: deceased; i. 0: disease-free/censored; 1: recurrence.
