# Supplementary material for: Reconstruction of lncRNA-miRNA-mRNA network based on competitive endogenous RNA reveals functional lncRNAs in skin cutaneous melanoma
Source: BMC Cancer. 2020 Sep 29;20:927. doi: 10.1186/s12885-020-07302-5 (PMC7523354; doi:10.1186/s12885-020-07302-5)
Supplement: Supplementary file 2 — Additional file 2: Supplementary Table 2. Differentially expressed miRNAs in GSE24996、GSE35579、GSE62372. [file 12885_2020_7302_MOESM2_ESM.docx]

| GSE24996 | GSE35579 | GSE62372 |
| --- | --- | --- |
| hsa-miR-363 | solexa-2683-338 | hsa-miR-125b |
| hsa-miR-202* | solexa-8048-104 | hsa-miR-101 |
| hsa-miR-454-3p | solexa-578-1915 | hsa-miR-100 |
| hsa-miR-142-5p | hsa-miR-663b | hsa-miRPlus-E1066 |
| hcmv-miR-UL148D | hsa-miR-675 | hsa-miRPlus-A1015 |
| hsa-miR-21 | hsa-miR-494 | hsa-miR-200c |
| hsa-miR-142-3p | hsa-miR-1273 | hsa-miR-125a-5p |
| hsa-miR-450 | hsa-miR-603 | hsa-miR-211 |
| hsa-miR-34c | hsa-miR-1285 | hsa-miRPlus-E1219 |
| hsa-miR-155 | hsa-miR-198 | hsa-miR-26a |
| hsa-miR-550 | hsa-miR-20b | hsa-miR-509-3p |
| hsa-miR-338 | hsa-miR-1248 | hsa-miR-26b |
| hsa-miR-20b | hsa-miR-1826 | hsa-miRPlus-E1202 |
| hsa-miR-22 | hsa-miR-519e* | hsa-miR-140-3p |
| hsa-miR-340 | solexa-826-1288 | hsa-miR-202 |
| hsa-miR-146b | hsa-miR-9 | hsa-miR-130a |
| hsa-miR-424 | hsa-miR-30c-1* | hsa-miR-1826 |
| hsa-miR-18a | solexa-5620-151 | SNORD38B-5 |
| hsa-miR-223 | hsa-miR-1300 | hsa-miR-205 |
| hsa-miR-106b | solexa-8211-102 | hsa-miRPlus-E1047 |
| hsa-miR-15a | hsa-miR-95 | hsa-miRPlus-E1290 |
| hsa-miR-130b | hsa-miR-639 | SNORD49A-5 |
| hsa-miR-625 | hsa-miR-9* | hsa-miR-203 |
| hsa-miR-30a-5p | hsa-miR-424 | hsa_SNORD4A |
| hsa-miR-339 | hsa-miR-886-3p | hsa-miR-1201 |
| hsa-miR-17-5p | hsa-miR-767-5p | hsa-let-7d |
| hsa-miR-29b | hsa-miR-192 | hsa-miR-23b |
| hsa-miR-15b | hsa-miR-566 | hsa-miRPlus-E1065 |
| hsa-miR-425-5p | solexa-4793-177 | hsa-let-7a |
| hsa-miR-19b | hsa-miR-576-3p | hsa-miRPlus-E1016 |
| hsa-miR-185 | hsa-miR-200b* | hsa-miRPlus-E1060 |
| hsa-miR-29a | hsa-miR-200b | hsa-let-7f |
| hsa-miR-106a | hsa-miR-183 | hsa-miR-21 |
| hsa-miR-362 | hsa-miR-200a | hsa-miR-24 |
| hsa-miR-205 | hsa-miR-182 |  |
| hsa-miR-211 | hsa-miR-203 |  |
| hsa-miR-200c | solexa-2580-353 |  |
| hsa-miR-149 | hsa-miR-205 |  |
| hsa-miR-204 | hsa-miR-224 |  |
| hsa-miR-99a | hsa-miR-149 |  |
| hsa-miR-455 | hsa-miR-455-5p |  |
| hsa-miR-200b | hsa-miR-455-3p |  |
| hsa-miR-125b | hsa-miR-141 |  |
| hsa-miR-193b | hsa-miR-378 |  |
| hsa-miR-768-3p | hsa-miR-99a |  |
| hsa-miR-489 | hsa-miR-195 |  |
| hsa-miR-100 | hsa-miR-876-3p |  |
| hsa-miR-224 | hsa-miR-888 |  |
| hsa-miR-378 | hsa-miR-1290 |  |
| hsa-miR-23b | hsa-miR-139-5p |  |
| hsa-miR-203 | hsa-miR-497 |  |
| hsa-miR-27b | hsa-miR-429 |  |
| hsa-let-7b | hsa-miR-154 |  |
| hsa-miR-564 | hsa-miR-1249 |  |
| hsa-let-7c | hsa-miR-200c |  |
| hsa-miR-125a | hsa-miR-218 |  |
|  | hsa-miR-204 |  |
|  | hsa-miR-140-5p |  |
|  | hsa-miR-214 |  |
|  | hsa-miR-891b |  |
|  | hsa-miR-574-3p |  |
|  | hsa-miR-125b-2* |  |
|  | hsa-miR-211 |  |
|  | hsa-miR-299-5p |  |
|  | hsa-miR-140-3p |  |
|  | hsa-miR-431 |  |
|  | hsa-miR-29b-2* |  |
|  | hsa-miR-885-5p |  |
|  | hsa-miR-365 |  |
|  | hsa-miR-27b* |  |
