# Supplementary material for: Reconstruction of lncRNA-miRNA-mRNA network based on competitive endogenous RNA reveals functional lncRNAs in skin cutaneous melanoma
Source: BMC Cancer. 2020 Sep 29;20:927. doi: 10.1186/s12885-020-07302-5 (PMC7523354; doi:10.1186/s12885-020-07302-5)
Supplement: Supplementary file 3 — Additional file 3: Supplementary Table 3. Univariate COX regression model for survival analysis of age, sex and stage. [file 12885_2020_7302_MOESM3_ESM.docx]

| Factors | Univariate COX regression model for survival analysis (OS, DFS) |
| --- | --- |
| Age | p < 0.001, p < 0.001 |
| Sex | p = 0.167, p = 0.198 |
| Stage | p < 0.001, p < 0.001 |
| OS: overall survival; DFS: disease-free survival | |
